# Integrated computational model of the bioenergetics of isolated lung mitochondria

**DOI:** 10.1371/journal.pone.0197921

**Published:** 2018-06-11

**Authors:** Xiao Zhang, Ranjan K. Dash, Elizabeth R. Jacobs, Amadou K. S. Camara, Anne V. Clough, Said H. Audi

**Affiliations:** 1 Department of Biomedical Engineering, Marquette University, Milwaukee, Wisconsin, United States of America; 2 Department of Biomedical Engineering, Medical College of Wisconsin, Milwaukee, Wisconsin, United States of America; 3 Department of Physiology, Medical College of Wisconsin, Milwaukee, Wisconsin, United States of America; 4 Zablocki V.A. Medical Center, Milwaukee, Wisconsin, United States of America; 5 Division of Pulmonary and Critical Care Medicine, Medical College of Wisconsin, Milwaukee, Wisconsin, United States of America; 6 Department of Anesthesiology, Medical College of Wisconsin, Milwaukee, Wisconsin, United States of America; 7 Department of Mathematics, Statistics, and Computer Science, Marquette University, Milwaukee, Wisconsin, United States of America; Stanford University, UNITED STATES

## Abstract

Integrated computational modeling provides a mechanistic and quantitative framework for describing lung mitochondrial bioenergetics. Thus, the objective of this study was to develop and validate a thermodynamically-constrained integrated computational model of the bioenergetics of isolated lung mitochondria. The model incorporates the major biochemical reactions and transport processes in lung mitochondria. A general framework was developed to model those biochemical reactions and transport processes. Intrinsic model parameters such as binding constants were estimated using previously published isolated enzymes and transporters kinetic data. Extrinsic model parameters such as maximal reaction and transport velocities were estimated by fitting the integrated bioenergetics model to published and new tricarboxylic acid cycle and respirometry data measured in isolated rat lung mitochondria. The integrated model was then validated by assessing its ability to predict experimental data not used for the estimation of the extrinsic model parameters. For example, the model was able to predict reasonably well the substrate and temperature dependency of mitochondrial oxygen consumption, kinetics of NADH redox status, and the kinetics of mitochondrial accumulation of the cationic dye rhodamine 123, driven by mitochondrial membrane potential, under different respiratory states. The latter required the coupling of the integrated bioenergetics model to a pharmacokinetic model for the mitochondrial uptake of rhodamine 123 from buffer. The integrated bioenergetics model provides a mechanistic and quantitative framework for 1) integrating experimental data from isolated lung mitochondria under diverse experimental conditions, and 2) assessing the impact of a change in one or more mitochondrial processes on overall lung mitochondrial bioenergetics. In addition, the model provides important insights into the bioenergetics and respiration of lung mitochondria and how they differ from those of mitochondria from other organs. To the best of our knowledge, this model is the first for the bioenergetics of isolated lung mitochondria.

## Introduction

In addition to the vital function of ATP production, mitochondria are involved in other important cellular functions, including apoptosis, calcium homeostasis, oxygen sensing, and nitric oxide signaling [1–5]. Mitochondria are also an important source of reactive oxygen species (ROS) under physiological and pathophysiological conditions, and are a primary target of oxidative stress [1, 2, 6]. Under normal conditions, mitochondria-derived ROS are involved in important signaling pathways, but when produced in excess, they contribute to oxidative stress, which is a key factor in the pathogenesis of lung diseases [2, 7].

Mitochondrial respiration accounts for 80–85% of total lung ATP, and glucose is the most prevalent oxidizable substrate in lungs under physiological conditions [8]. There is ample evidence that mitochondrial dysfunction plays a key role in the pathogenesis of lung diseases, including acute lung injury (ALI), which is one of the most common causes of admission to intensive care units [2]. Using animal models of ALI and its most severe form, acute respiratory distress syndrome (ARDS), previous studies have reported significant changes in different mitochondrial processes, including decreases in the activity of complexes I and II, decreases in TCA cycle enzyme activities, dissipation of membrane potential, and impairment of ATP generation [9–14]. In addition, alteration in lung mitochondrial bioenergetics has been linked to pulmonary edema formation, which is a cardinal feature of ALI/ARDS [8]. Bongard et al. demonstrated that the integrity of the pulmonary endothelial barrier is dependent on normal lung mitochondrial bioenergetics [10].

There is a wealth of information regarding the impact of lung injury on mitochondrial enzymes, transporters and macromolecules [10, 15]. However, because of the interdependence of mitochondrial processes, measurement of a change in one or more mitochondrial processes in response to injury is not sufficient to predict the functional impact of this change on overall mitochondrial bioenergetics. Integrated computational modeling provides a mechanistic and quantitative framework for describing mitochondrial bioenergetics, for quantifying the impact of a change in one or more processes on overall mitochondrial bioenergetics, and for identifying potential mitochondrial targets for diagnostic, prognostic, and therapeutic purposes. This type of modeling has been used to describe mitochondrial bioenergetics of other organs, including heart and skeletal muscle under physiological and pathophysiological conditions [16–18]. However, to the best of our knowledge, no such model exists for bioenergetics of isolated lung mitochondria.

Therefore, the objective of this study was to use new and published experimental data to develop and validate a thermodynamically-constrained integrated computational model of the bioenergetics of isolated lung mitochondria. Key results of this study are 1) development and validation of the first integrated computational model of the bioenergetics of isolated lung mitochondria, 2) coupling of this integrated model to a pharmacokinetics model of the mitochondrial uptake and release of the cationic membrane potential probe rhodamine 123 during different respiratory states for quantifying mitochondrial membrane potential, and 3) model simulations that provide important insights into the bioenergetics and respiration of isolated lung mitochondria and how they differ from those of mitochondria isolated from other organs.

## Materials and methods

### Experimental methods

All chemicals were purchased from Sigma-Aldrich (St. Louis, MO), unless stated otherwise. All treatment protocols and procedures for animals were approved by the Institutional Animal Care and Use Committees of the Zablocki Veterans Affairs Medical Center, the Medical College of Wisconsin, and Marquette University.

### Isolation of lung mitochondria

Adult male Sprague-Dawley rats (320-360g) were anesthetized (pentobarbital sodium 50–100 mg/kg, ip) and the lungs were rapidly exposed and cleared of residual blood with 50 ml cold perfusion solution (physiologic saline buffered with 10 mM HEPES, pH 7.4, and containing 5.5 mM glucose) via the right ventricle. The lungs were then removed from the chest, and the trachea, large airways and large vessels were removed, after which the peripheral lung was placed in an ice-cold homogenization buffer (pH 7.4) containing 10 mM HEPES, 200 mM mannitol, 70 mM sucrose, 1 mM EGTA, 2% fatty acid-free BSA, and protease inhibitor cocktail Set III (50 μl/g lung tissue; Calbiochem, San Diego, CA) and minced over ice. Lung tissue was then homogenized using a Teflon pestle. The resulting homogenate was then centrifuged (Sorvall Superspeed RC-5B, Norwalk, CT) at 2,000 × g and 4°C for 15 minutes. The supernatant was transferred to a clean tube and centrifuged at 17,800 × g at 4°C for 15 minutes. The resulting supernatant was discarded and the remaining pellet was resuspended in 5 ml ice-cold homogenization solution and centrifuged at 17,800 × g at 4°C for 15 minutes. The supernatant was discarded and the final pellet was resuspended in 0.3–4 ml ice-cold buffer (same as the homogenization buffer without BSA or the protease inhibitor cocktail) and stored on ice to be used for the respirometry and membrane potential studies described below. Mitochondrial protein was determined using the Pierce BCA protein assay with bovine serum albumin as the standard. Mitochondrial yield was ~2 mg/rat lung.

### Mitochondrial oxygen consumption

Mitochondrial oxygen consumption (respiration) was measured polarographically at 23°C with a Strathkelvin 782 2-Channel Oxygen System (Strathkelvin Instrument). Briefly, 0.55 ml respiration buffer (130 mM KCL, 5 mM K_2_HPO_4_, 20 mM MOPS, 0.1 mM EDTA, 0.001 mM Na_4_P_2_O_7_ and 0.1% BSA, pH 7.2) was transferred to the reaction chamber. After 2 minutes, 0.55 mg mitochondrial protein (1 mg/ml) was transferred to the 0.55 ml reaction chamber. Stock solution containing respiratory substrates (pyruvate (10 mM) + malate (5 mM), glutamate (10 mM) + malate (5 mM), or succinate (7 mM) at pH 7.2) was introduced into the reaction chamber using a Hamilton syringe. Once the oxygen consumption rate reached steady state (state 2), ADP (100 µM or 50 µM) was added to stimulate the rate of oxygen utilization (state 3). State 4 was measured as the rate of oxygen consumption following depletion of ADP in the reaction chamber. The respiratory control index (RCI) for pyruvate + malate, malate + glutamate, or succinate was then calculated as the ratio of state 3 and state 4 respiratory rates. The rate of uncoupled oxygen consumption was measured in the presence of the uncoupler carbonyl cyanide-4-(trifluoromethoxy) phenylhydrazone (FCCP, 2 µM).

For a subset of experiments, maximal complex IV activity was measured at 30°C using the complex IV substrate tetramethyl-p-phenylenediamine (TMPD, 0.3 mM) as a direct artificial electron donor in the presence of antimycin A (1 µM, complex III inhibitor). Ascorbate (1.3 mM) was also added to the medium to maintain TMPD in its reduced form. Furthermore, the integrity of the mitochondrial outer-membrane was assessed by evaluating the ability of exogenous cytochrome c (10 µM) added to the medium to stimulate oxygen consumption.

#### Mitochondrial membrane potential

As previously described [19], mitochondrial membrane potential (ΔΨ_m_) studies were performed at room temperature (23°C) using a Photon Technology International (PTI) QuantaMaster spectrofluorometer (HORIBA Scientific, Edison, New Jersey) that monitored and recorded the R123 fluorescent signal (503/527 nm excitation/emission wavelength) continuously over time. Briefly, a cuvette containing 1 ml of the respiration buffer (pH 7.2), R123 (200 nM), and either pyruvate (10 mM) + malate (5 mM) or succinate (7 mM) as respiratory substrate was placed in the cuvette holder of the spectrofluorometer. After 2 minutes, mitochondrial protein (1 mg/ml) was added, and then once the R123 fluorescent signal had reached steady-state (state 2), ADP (100 µM or 50 µM) was added to evaluate the ADP-stimulated depolarization of ΔΨ_m_. The maximum uncoupled ΔΨ_m_ was then determined by adding FCCP (2 µM). Since the R123 medium concentrations are low (≤ 200 nM), quenching effects are negligible [20]. Hence, the R123 fluorescent signal was converted to R123 concentration by assuming a linear relationship between R123 medium concentration and fluorescent signal.

#### Model development

**Fig 1:**
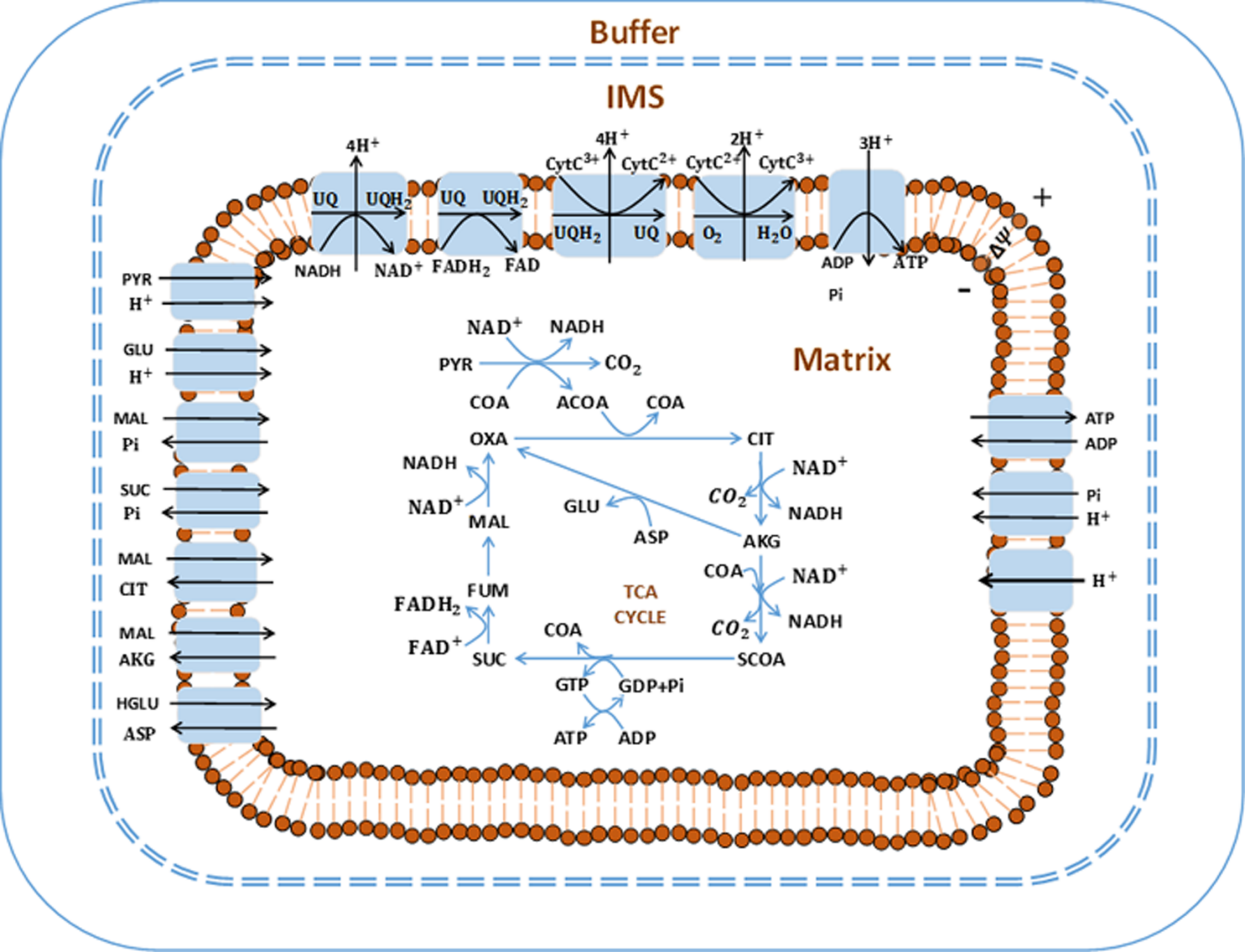
Structure of the isolated lung mitochondrial bioenergetics model. The model consists of three regions (extra-mitochondrial buffer, mitochondrial matrix, and inter-membrane space (IMS)). Major reactions include TCA cycle reactions and electron transport chain reactions. Transport exists between mitochondrial matrix and IMS. Most of the metabolites are assumed to be freely permeable across mitochondria outer-membrane. The major biochemical species included in this model are: PYR: pyruvate, CoA: coenzyme-A, ACoA: acetyl-CoA, OXA: oxaloacetate, CIT: citrate, AKG: a-ketogluterate, SCoA: succinyl-CoA, SUC: succinate, FUM: fumarate, MAL: malate, GLU: glutamate, and ASP: aspartate, NAD and NADH: oxidized and reduced form of nicotinamide adenine dinucleotide, respectively, FAD and FADH_2_: oxidized and reduced form of Flavin adenine dinucleotide, respectively, ADP and ATP: adenosine triphosphate and adenosine diphosphate, respectively, UQ and UQH_2_: oxidized and reduced form of ubiquinone, respectively, CtyC^3+^ and CytC^2+^: oxidized and reduced form of cytochrome c, respectively.

In what follows, all figure and table numbers with prefix “A” are found in S1 Supporting Information. The integrated model of the bioenergetics of isolated lung mitochondria was developed based on the thermodynamic and kinetic properties of the mitochondrial metabolic reactions and transporters [17, 18, 21]. As shown in Fig 1, the model consists of three regions: the extra-mitochondrial region (buffer), the mitochondrial matrix region, and the inter-membrane space (IMS) region. Moreover, the model accounts for the dynamics of forty state variables, including thirty-seven metabolite concentrations in the matrix and buffer regions, two ion concentrations (matrix H^+^ and buffer H^+^ concentrations), and mitochondrial membrane potential (ΔΨ_m_). Volumes of the three regions along with initial concentrations of major metabolites are given in Table 1.

**Table 1:** General model parameter values*.

Parameter	Value	Source
Mitochondria matrix volume (*V*_m_)	1 µl/mg mitochondria protein	[18, 22, 23]
Mitochondria IMS volume (*V*_ims_)	Assume to be 10% of *V*_m_	
Total cytochrome c content	0.33 nmol/mg mitochondria	[24]
Total pyridine nucleotide content (NAD + NADH)	1.73 nmol/mg mitochondria	[24]
Total flavin adenine dinucleotide concentration (FAD + FADH2)	0.7 mM	[25]
Total adenine nucleotide content (ATP and ADP)	6.4 nmol/mg mitochondria	[24]
Total coenzyme A content (SCoA + ACoA + CoA)	0.93 nmol/mg mitochondria	[24]
Aspartate + glutamate concentration in mitochondria matrix	12 mM	[26]
Total ubiquinone concentration (UQ + UQH_2_)	0.52 mM	[23]

* Content is converted to concentration by using 1 µl/mg mitochondria water content.

Fifteen reaction fluxes are included in this integrated model, including ten reactions in the tricarboxylic acid cycle (TCA or Krebs cycle) and five reactions in the respiratory system (complex I-complex V). Unlike liver mitochondria and kidney mitochondria, which can produce oxaloacetate (OXA) from pyruvate, pyruvate carboxylase activity is extremely low in lung mitochondria [27]. Thus, pyruvate carboxylase is not included in this model. In isolated mitochondrial experiments, bovine serum albumin (BSA) was used to bind free fatty acids and their esters [28]. Therefore, fatty acid oxidation was not considered in this isolated mitochondrial model. All the reactions in the integrated bioenergetics model are listed in Table A2 in S1 Supporting Information.

Additionally, the model includes ten transport processes to account for the exchange of key metabolic species between the mitochondrial matrix and IMS regions. The mitochondrial inner membrane is impermeable to most metabolites. Therefore, except for proton leak, the exchange of species between the mitochondrial matrix and IMS regions is assumed to be catalyzed by specific transporters, such as inorganic phosphate carrier (PIC) and adenine nucleotide translocase (ANT). The proton leak between the IMS and mitochondrial matrix regions is modeled using modified Goldman-Hodgkin-Katz equation [18]. All the transporters in the model are listed in Table A3 in S1 Supporting Information.

The mitochondrial outer membrane has porins that allow free transport of small molecules [18]. Therefore, we assumed that the concentration differences of most of the metabolic species between the IMS and extra-mitochondrial space to be negligible. However, cytochrome c, which has a molecular weight of about 12 KD, and hence too large to pass through the mitochondrial outer membrane under normal conditions, was assumed to exist only in the IMS region.

#### Reaction and transport flux expressions

In the proposed integrated bioenergetics model, all enzymatic reactions are assumed to follow a generalized random-ordered rapid-equilibrium kinetic mechanism [29], which encompass all possible kinetic mechanisms as special cases. Thus, for each reaction, all pertinent substrates must bind to the enzyme together before the catalysis can take place. For a general multi-substrate and multi-product enzymatic reaction:

$$\overset{N_{s}}{\underset{i=1}{\sum }}\alpha_{i}S_{i}↔\overset{N_{P}}{\underset{j=1}{\sum }}\beta_{j}P_{j}$$ (1)

where *S*_*i*_ is i^th^ substrate, *P*_*j*_ is j^th^ product, *N*_*s*_ and *N*_*p*_ are the number of substrates and products, respectively, and *α*_*i*_ and *β*_*j*_ are the corresponding stoichiometric coefficients. The general form of the reaction flux, *J*, accounting for the thermodynamic (Haldane) constraint is given by:

$$J=\frac{\frac{V_{maxf}}{\prod_{i=1}^{N_{s}}K_{S_{i}}{}^{\alpha_{i}}}\left(\prod_{i=1}^{N_{s}}{\left[S_{i}\right]}^{\alpha_{i}}-\frac{\prod_{j=1}^{N_{p}}{\left[P_{j}\right]}^{\beta_{j}}}{{K^{\prime }}_{eq}}\right)}{\prod_{i=1}^{N_{s}}\left(1+\frac{{\left[S_{i}\right]}^{\alpha_{i}}}{K_{S_{i}}{}^{\alpha_{i}}}\right)\times \prod_{j=1}^{N_{p}}\left(1+\frac{{\left[P_{j}\right]}^{\beta_{j}}}{K_{P_{j}}{}^{\beta_{j}}}\right)}$$ (2)

where *K*_*Si*_ and *K*_*Pj*_ are binding constants corresponding to substrates and products, respectively; [*S*_*i*_] and [*P*_*j*_] are concentrations of substrate *i* and product *j*, respectively; *V*_*maxf*_ is the maximum forward reaction rate; and *K′*_*eq*_ is the apparent equilibrium constant for the reaction, which is the value of the equilibrium-state reaction quotient (i.e. ratio of product of product concentrations over product of substrate concentrations) at specified thermodynamic conditions (i.e. temperature, ionic strength and pH). Detailed derivations of this general reaction flux equation and its various special cases are included in S1 Supporting Information.

In the presence of cofactor pairs (e.g. NADH and NAD^+^, ATP and ADP, GTP and GDP, CoA and ACoA, or CoA and SCoA), the generalized reaction flux Eq (2) can be modified appropriately so as to not include any interactive cofactor product terms [29]. The assumption is that the substrate and product represented as a cofactor pair bind with a given enzyme at the same binding site, in which case the resulting reaction flux equation does not include the corresponding substrate and product multiplication terms in the denominator of Eq 2. Eq 2 can also be suitably modified to account for other reaction kinetic mechanisms (e.g. sequential-ordered mechanisms, ping-pong mechanisms). The flux expressions of all the enzymatic reactions are included in the S1 Supporting Information.

In this integrated bioenergetics model, all the transport processes across the mitochondrial inner membrane, with the exception of the proton leak, are catalyzed by specific metabolite transporters involving two different species (co-transporters and anti-porters). For generality, a random-ordered rapid-equilibrium binding mechanism is assumed for all the transporters, i.e., the transporter can bind to the two species in an arbitrary order, similar to that described for a reversible two-substrate two-product enzymatic reaction. Thus, the general form of the transport flux expression is assumed to be the same as that for the reaction flux expression (i.e. Eq 2). The flux expressions for all the metabolite transporters are included in S1 Supporting Information.

#### Accounting for the effect of pH on equilibrium constants

The proposed integrated bioenergetics model accounts for the pH dependence of the apparent equilibrium constants for proton-releasing reactions (Eq 3) and for proton-consumption reactions (Eq 4) [17, 23, 30]:

$${K^{\prime }}_{eq}={K^{\prime }}_{eq}^{0}\times 10^{pH-7}=e^{-\Delta_{r}{G^{\prime }}_{}^{0}/RT}\times 10^{pH-7}$$ (3)

$${K^{\prime }}_{eq}={K^{\prime }}_{eq}^{0}\times 10^{7-pH}=e^{-\Delta_{r}{G^{\prime }}_{}^{0}/RT}\times 10^{7-pH}$$ (4)

where ${K^{\prime }}_{\text{eq}}^{0}$ is the reaction apparent equilibrium constant (${K^{\prime }}_{eq}$) at pH of 7, and Δ_r_*G*^'0^, *R* and *T* are the standard Gibbs free energy of the reaction at pH = 7, gas constant, and temperature, respectively.

#### Accounting for the effect of temperature on enzymatic reaction and transport rates

To allow for model simulations at different temperatures, we account for the temperature effects on the maximal reaction and transport rates (*V*_*max*_’s and *T*_*max*_’s) using the Q_10_ temperature coefficient [23].

$$R_{2}/R_{1}=Q_{10}{}^{(T_{2}-T_{1})/10}$$ (5)

where *T* is the temperature, *R*_*2*_/*R*_*1*_is the correction coefficient (i.e. the ratio of maximal reaction and transport velocities at temperature *T*_*2*_ and *T*_*1*_), and *Q*_*10*_ is the temperature coefficient which was set to 2.5.

#### Governing ordinary differential equations for the integrated bioenergetics model

The governing ordinary differential equations describing the dynamic changes in the concentrations (*C*) of the forty biochemical species were derived based on the principle of mass balance. The change in the concentration of a given species within the mitochondria matrix region and extra-mitochondria (buffer) region are given by:

$$V_{m}\frac{dC_{m,j}}{dt}=\sum \alpha_{m,\;j}J_{m,j}-\sum J_{m-e,j}$$ (6)

$$\;\;\;\;V_{e}\frac{dC_{e,j}}{dt}=\sum J_{m-e,j}$$ (7)

where *J*_*mj*_ is the j^th^ reaction flux in the mitochondrial matrix and *J*_*m-e*,*j*_ is the j^th^ transport flux between mitochondria matrix region and extra-mitochondria buffer region, *V*_*m*_ and *V*_*e*_ are the volumes of the mitochondrial matrix region and the extra-mitochondria region, respectively. For the extra-mitochondria region which does not include chemical reactions, the right hand side of Eq 7 contains just transport fluxes. Detailed mass balance equations are included in S1 Supporting Information.

The rate of change of mitochondrial membrane potential (ΔΨ_m_) is given by Eq 8 [18]:

$$\frac{d\Delta \Psi_{m}}{dt}=\frac{1}{C_{imm}}\left(4J_{CI}+2J_{CIII}+4J_{CIV}-3J_{CV}-J_{HLEAK}-J_{ANT}\right)$$ (8)

where *C*_*imm*_ is the capacitance of the inner mitochondrial membrane; *J*_*CI*_, *J*_*CIII*_, *J*_*CIV*_ and *J*_*CV*_ are the reaction fluxes of complex I, complex III, complex IV and complex V, respectively;*J*_*Hleak*_ is the proton leak between the IMS and mitochondria matrix regions; and *J*_*ANT*_ is the transport flux characterizing ATP/ADP exchange via the ANT.

MATLAB function ‘*ode15s*’ (MathWorks Inc.) was used to solve the system of governing differential equations.

### Methods used for estimating the values of the unknown model parameters

As described below, some model parameters were set at published values, while others were estimated from new and published data, including isolated enzyme and transporter kinetics (see S1 Supporting Information), TCA cycle kinetics [27], and respirometry [24, 27].

#### Estimation of the intrinsic model parameters

The binding constants (*K’*s) of various substrates and products for enzymes and transporters were either set to published values [18, 31] or estimated using published kinetic data from isolated mitochondrial enzymes and transporters. Some of the isolated enzyme and transporter kinetic data were from heart, kidney or liver mitochondria due to the scarcity of such data from lungs. The assumption is that the binding constants are intrinsic model parameters, and hence their values should be organ-independent. For a given enzyme or transporter, the MATLAB function ‘*fmincon*’, a nonlinear program solver, was used with a least-squares objective function to estimate the values of the *K’*s from the published kinetic data for the enzyme or transporter by fitting a given enzyme or transporter model to pertinent kinetic data. Models of reaction and transport fluxes for various enzymatic reactions and metabolite transporters, parameter estimation results, as well as the kinetic data are included in S1 Supporting Information.

#### Estimation of the extrinsic model parameters

With the binding constants (*K’s*) of the various enzymes and transporters known, a genetic algorithm (GA, MATLAB function ‘*ga*’) was then used to estimate the values of the extrinsic model parameters (i.e. *V*_*maxf*_s and *T*_*maxf*_s) of the integrated bioenergetics model that best fit new and published dynamic kinetic data obtained from isolated lung mitochondria, including TCA cycle kinetics data [27], and respirometry data at different respiratory states [24, 27]. Thus, the resulting values of extrinsic model parameters are specific to isolated rat lung mitochondria. GA is a global parameter estimation algorithm that mimics the process of natural selection and can escape local minima [32]. For the integrated model extrinsic parameter estimation, the objective function ƒ used is:

$$f={\sum_{j=1}^{M}\frac{1}{N}\overset{N}{\underset{i=1}{\sum }}\left(\frac{x_{i,j}-X_{i,j}}{X_{i,j}}\right)}^{2}$$ (9)

where *x*_*i*,*j*_ and *X*_*i*,*j*_ are the model solutions and the corresponding experimental data at the i^th^ time point and j^th^ data set, respectively. *N* is number of data points and *M* is the number of data sets used for the parameter estimation.

### Pharmacokinetic model for the mitochondrial uptake and release of the cationic dye rhodamine 123 (R123)

To validate the integrated bioenergetics model, we assessed its ability to predict experimental data that were not used for estimating the values of the unknown model parameters. To that end, we measured mitochondrial membrane potential (ΔΨ_m_) using the fluorescent cationic dye R123 in isolated rat lung mitochondria. The fluorescent intensity was converted to dye concentration and compared with model simulations under the same experimental conditions.

To simulate R123 dye disposition in isolated mitochondria, the integrated bioenergetics model was coupled with a modified version of a pharmacokinetic model by Gan et al. for the cellular uptake and mitochondrial accumulation of R123 [33]. Two additional state variables were needed, namely R123 concentrations in the mitochondria matrix [*R*123]_*m*_ and in the extra-mitochondrial buffer[*R*123]_*e*_. The process which accounted for the transport of R123 from the buffer to the matrix driven by an electrochemical gradient was modeled using the Goldman-Hodgkin-Katz equation [20].

$$J_{R123}=p\frac{zF}{RT}\left(\frac{{\left[R123\right]}_{e}e^{zF/\text{RT}}-{\left[R123\right]}_{m}}{e^{zF/\text{RT}}-1}\right)$$ (10)

where *F* is Faraday’ s constant, *z* is R123 valence, *R* is the gas constant, *T* is temperature, and *p* is the permeability coefficient of the membrane which was set to 1.38 mol/(liter mitochondria)/s/M.

The ordinary differential equations that describe the change in the concentrations of R123 in matrix and buffer are:

$$V_{m}^{APP}\frac{d{\left[R123\right]}_{m}}{dt}=-V_{e}^{APP}\frac{d{\left[R123\right]}_{e}}{dt}=J_{R123}$$ (11)

where $V_{m}^{APP}$ and $V_{e}^{APP}$ are the apparent volumes of mitochondrial matrix region (*V*_*m*_) and extra-mitochondria buffer region (*V*_*e*_), respectively [33].

## Results

### Estimated values of the unknown model parameters

Most of the intrinsic parameters of the integrated bioenergetics model, such as the binding constants (*K’*s) of the enzymatic reactions and transport processes, were estimated based on previously published isolated enzyme or transporter kinetic data shown in Figs A5–A14 in S1 Supporting Information. For instance, the parameters in Table A4 in S1 Supporting Information were estimated by fitting Eq A16 in S1 Supporting Information to the data in Fig A5 in S1 Supporting Information using the MATLAB function “*fmincon*”. The solid lines superimposed on the data in Figs A5–A14 in S1 Supporting Information are fits of corresponding enzyme/transporter models to the data in S1 Supporting Information. Since the binding constants of the enzymatic reactions and transport processes are intrinsic model parameters, they are assumed to be tissue-independent [23]. It is worth noting that the extrinsic model parameter *T*_*maxf*_s and *V*_*maxf*_s values estimated from the isolated enzyme and transporter kinetic data were not used in the integrated bioenergetics model since those parameters are expected to be dependent on the tissue source of mitochondria.

**Fig 2:**
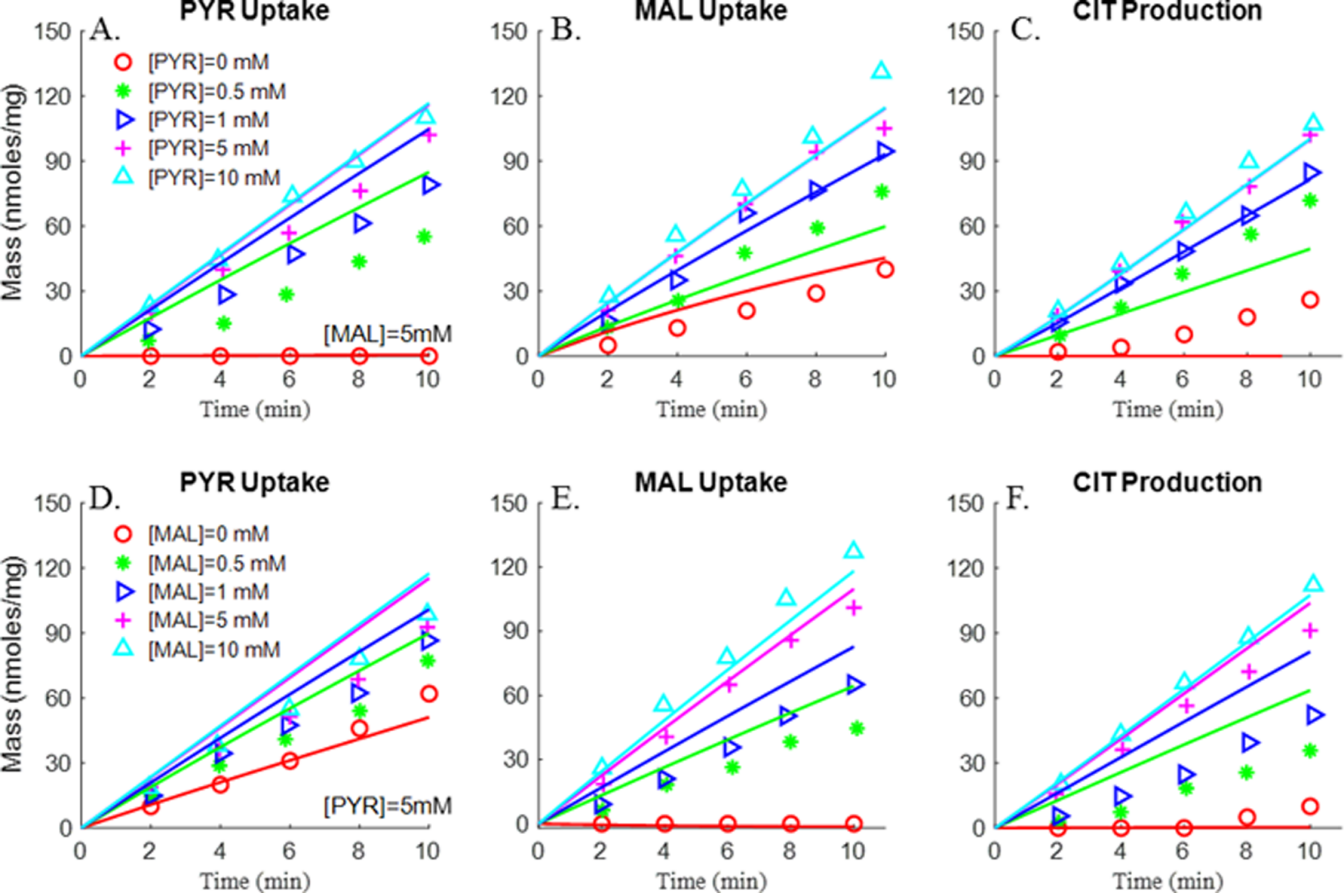
Substrate-dependent responses of pyruvate (PYR) uptake, malate (MAL) uptake and citrate (CIT) production (Evans et al. [27]). Rat lung mitochondria were incubated at 30°C in 1.0 ml buffer containing 4 mM Pi, and 0.7 mg mitochondria protein, buffer pH = 7.4. Mitochondria were incubated in the presence of different substrate concentrations. Fig 2A, 2B and 2C: (A) mitochondria were incubated with 5 mM MAL ([MAL] = 5 mM) and different concentrations of PYR: [PYR] = 0 mM (red circle), [PYR] = 0.5 mM (green star), [PYR] = 1 mM (blue triangle), [PYR] = 5 mM (purple plus sign), [PYR] = 10 mM (cyan triangle). Figs 2D, 2E and 1F: mitochondria were incubated with fixed PYR concentration ([PYR] = 5 mM) and different concentrations of MAL: [MAL] = 0 mM (red circle), [MAL] = 0.5 mM (green star), [MAL] = 1 mM (blue triangle), [MAL] = 5 mM (purple plus sign), [MAL] = 10 mM (cyan triangle). PYR uptake rate (A, D), MAL uptake rate (B, E) and CIT production rate (C, F) were measured over a 10-minute incubation period. Symbols are experimental data and lines are integrated model fits.

**Fig 3:**
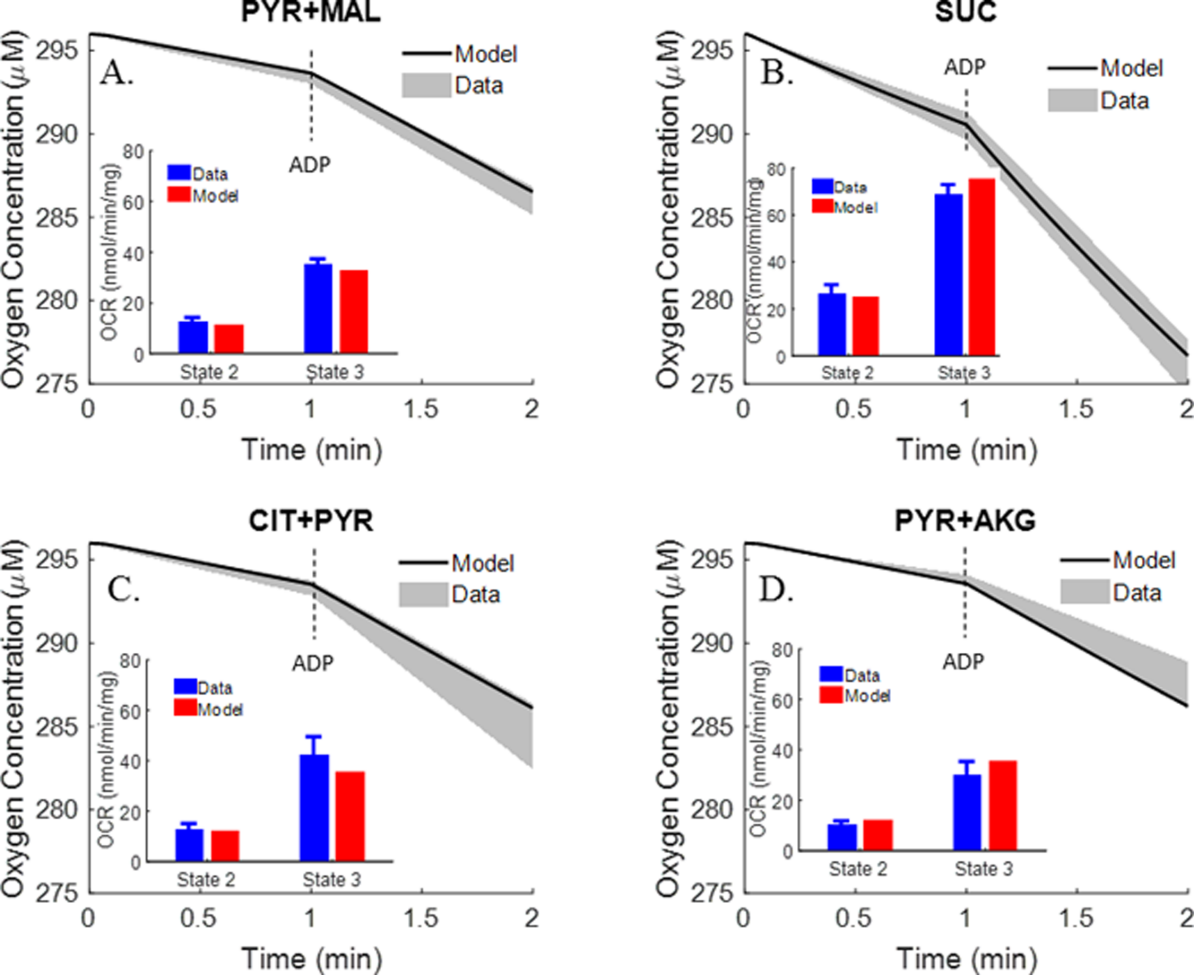
Respirometry data (30°C) (Evans et al. [27]). Rat lung mitochondria were incubated at 30°C in 1.9 ml buffer containing 4 mM Pi, and 0.7 mg mitochondria protein, buffer pH = 7.4. Oxygen consumption rates (OCR, nmol/min/mg) were calculated under state 2, state 3 conditions. Different metabolic substrates were used: (A) PYR + MAL, (B) SUC, (C) CIT + PYR and (D) PYR + AKG. Shaded areas are experimental data (mean ± SE) and solid lines are integrated model fits to the data. In the inset figures, the same experimental data are plotted as blue bars (mean ± SE) and the integrated model fits are plotted as red bars.

**Fig 4:**
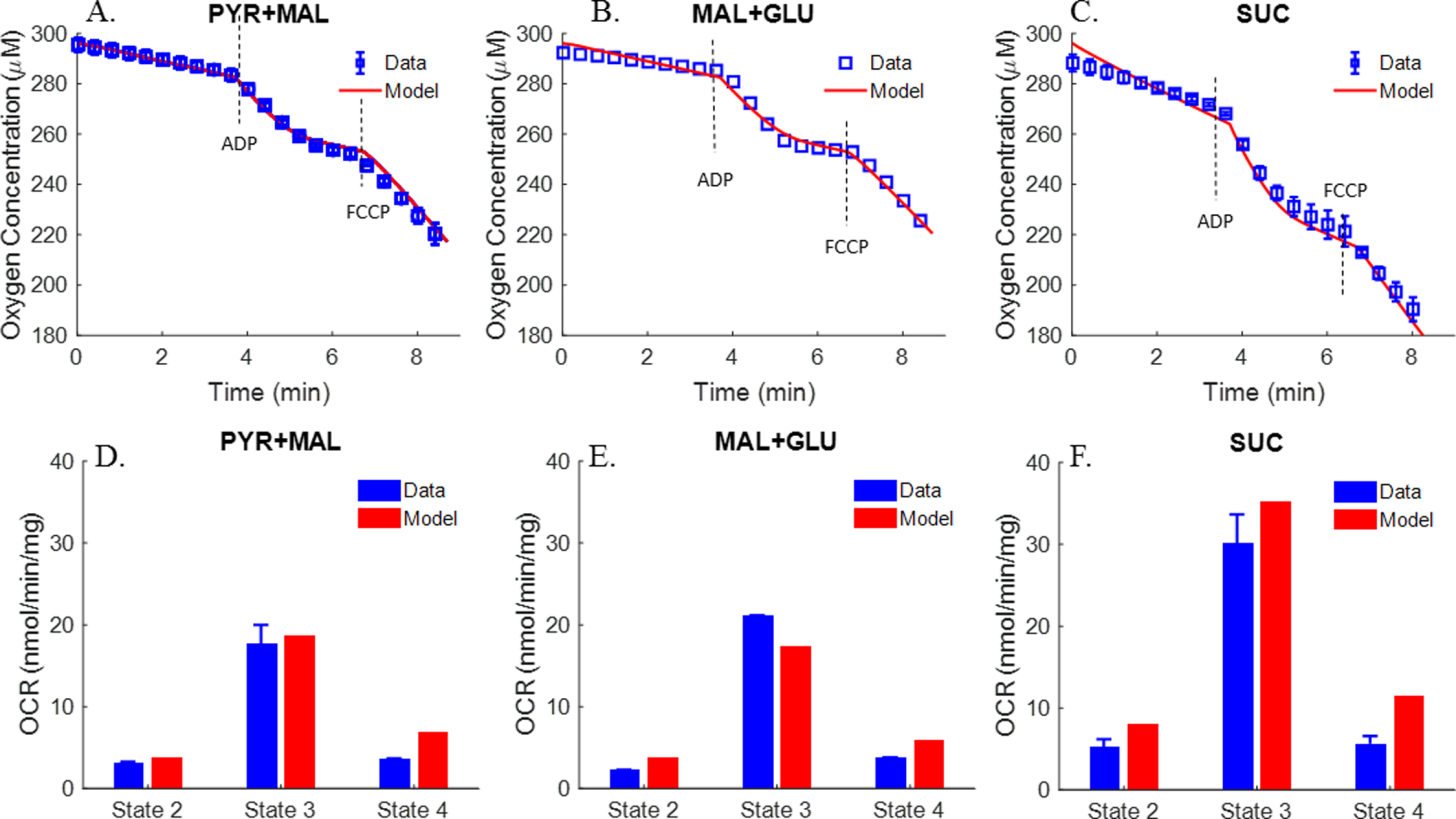
Respirometry data at room temperature (23˚C). Rat lung mitochondria were incubated in 0.55 ml buffer containing 2 mM Pi, and 0.55 mg mitochondria protein, buffer pH = 7.2. Average oxygen concentrations in buffer are shown under state 2, state 3, state 4 and uncoupled state 3 using pyruvate (PYR) + malate (MAL) (n = 4, panel A), MAL + glutamate (GLU) (n = 1, panel B), or succinate (SUC) (n = 4, panel C) as substrates. ADP and FCCP was added to buffer at 3.5 min and 6.5 min, respectively. Symbols are experimental data (mean ± SE) and solid lines are integrated model fits to the data. For the integrated model fits to the uncoupled state 3 data, the estimated value of the proton leak parameter *T*_*maxf*, *LEAK*_ in Table 2 was increased by seven folds. Panels D, E, and F show average oxygen consumption rates (OCR, nmol/min/mg) under states 2, 3 and 4 using PYR + MAL (n = 4), MAL + GLU (n = 1), or SUC (n = 4) as substrates. Blue bars are experimental data (mean ± SE) and red bars are integrated model fits to the data.

With the *K’*s of the various enzymes and transporters known, the next step was to estimate the extrinsic parameters (i.e. *T*_*maxf*_s and *V*_*maxf*_s) of the integrated bioenergetics model using kinetic data from isolated rat lung mitochondria. Multiple datasets form isolated rat lung mitochondria were used, including previously published TCA cycle metabolite uptake/release dynamic data (Fig 2) and respirometry data at 30°C (Fig 3) by Evans et al. [27], and newly measured respirometry data at room temperature (23°C, Fig 4). All of the extrinsic parameters (Table 2) of the integrated bioenergetics model were estimated by simultaneously fitting the integrated model solution to kinetic data from isolated rat lung mitochondria (Figs 2–4) using genetic algorithm (MATLAB function “*ga*”). The estimated values of the extrinsic parameters of the integrated bioenergetics model are shown in Table 2.

**Table 2:** Estimated values of the extrinsic parameters of the integrated model (30°C).

Parameters	Definition	Value (nmol/min/mg)
*V_maxf, PDH_*	Maximum forward reaction rate of PDH	307
*V_maxf, CITS_*	Maximum forward reaction rate of CITS	4631
*V_maxf, CITDH_*	Maximum forward reaction rate of CITDH	531
*V_maxf, AKGDH_*	Maximum forward reaction rate of AKGDH	780
*V_maxf, SCAS_*	Maximum forward reaction rate of SCAS	5816
*V_maxf, NDK_*	Maximum forward reaction rate of NDK	4.33×10^6^
*V_maxf, SUCDH_*	Maximum forward reaction rate of SDH	1.08×10^4^
*V_maxf, FH_*	Maximum forward reaction rate of FH	6.4×10^3^
*V_maxf, MDH_*	Maximum forward reaction rate of MDH	3.3×10^3^
*V_maxf, GOT_*	Maximum forward reaction rate of GOT	975
*V_maxf, CI_*	Maximum forward reaction rate of CI	11
*V_maxf, CII_*	Maximum forward reaction rate of CII	220
*V_maxf, CIII_*	Maximum forward reaction rate of CIII	2.23×10^4^
*V_maxf, CIV_*	Maximum forward reaction rate of CIV	0.27
*V_maxf, CV_*	Maximum forward reaction rate of CV	589
*T_maxf, DCC(SUC)_*	Maximum transport rate of DCC(SUC)	1699
*T_maxf, DCC(MAL)_*	Maximum transport rate of DCC(MAL)	22
*T_maxf, OME_*	Maximum transport rate of OME	1
*T_maxf, TCC_*	Maximum transport rate of TCC	81.3
*T_maxf, PYRH_*	Maximum transport rate of PYRH	96.6
*T_maxf, PIC_*	Maximum transport rate of PIC	2.4×10^4^
*T_maxf, ANT_*	Maximum transport rate of ANT	523.9
*T_maxf, GLUH_*	Maximum transport rate of GLUH	5
*T_maxf, GAE_*	Maximum transport rate of GAE	646
*T_maxf, LEAK_*	Maximum rate of proton leak	36

As shown in Fig 2, mitochondrial pyruvate uptake, malate uptake, and citrate production/release were measured in isolated rat lung mitochondria by Evans et al. in the presence of 5 mM malate and varying pyruvate concentrations (top panel) or 5 mM pyruvate and varying malate concentrations (bottom panel) in buffer [27]. Results show that pyruvate uptake, malate uptake, and citrate release increased with increasing buffer pyruvate concentration at 5 mM of malate (top panel) and increasing malate concentration at 5 mM of pyruvate (bottom panel), and that those flux rates saturated when pyruvate and malate concentrations were greater than 5 mM.

In addition to the TCA cycle metabolite uptake/release data, the study by Evans et al. also provided mitochondrial oxygen consumption rates using different metabolic substrates [27]. As shown in Fig 3, mitochondria oxygen consumption rates under states 2 and 3 were measured in the presence of different complex I substrates (pyruvate + malate, α-ketoglutarate (AKG) + pyruvate, and citrate + pyruvate) and complex II (succinate) substrates, with the buffer temperature set at 30°C [27]. The data show that states 2 and 3 oxygen consumption rates tended to be higher in the presence of complex II substrate as compared to those in the presence of complex I substrates. However, only oxygen consumption rates are provided in this study, whereas key information such as length of state 3 and oxygen dynamic changes under state 3 conditions are lacking. Thus for additional information on rat lung mitochondria electron transport chain (ETC) for the estimation of pertinent extrinsic model parameters, we measured oxygen consumption (Fig 4) at 23°C using pyruvate + malate or glutamate + malate as complex I substrates or succinate as complex II substrate. The resulting average oxygen concentration kinetic data under states 2, 3 and 4 were used along with the data in Figs 2 and 3 for estimating the extrinsic parameters of the integrated bioenergetics model. The uncoupled state 3 kinetic data in Fig 4 were simulated by increasing the estimated value of the proton leak parameter *T*_*maxf*, *LEAK*_ in Table 2 by seven folds.

Figs 3 and 4 show different state 2 respiration rates. Since state 2 respiration rates in Fig 4 are ~40% (after accounting for temperature effect) of those in Fig 3 (Evans et al. data), the maximum rate of proton leak (*T*_*maxf*, *LEAK*_) was adjusted to account for those differences. Therefore, for the data in Fig 4, *T*_*maxf*, *LEAK*_ was set at 40% of the value used to fit the data in Fig 3 (Table 2). However, for experimental data from the same study (Figs 2 and 3), *T*_*maxf*, *LEAK*_ was set to the same value.

### Measures of identifiability and estimatility of the extrinic parameters of the integrated model

To assess the identifiability and estimability of the extrinsic parameters of the integrated bioenergetics model (Table 2), we estimated the parameters’ normalized sensitivity coefficients and a matrix of correlation coefficients between the model parameters. The normalized sensitivity coefficients provide information about the contribution of each of the extrinsic model parameters to the overall model solution, whereas the correlation coefficient matrix provides information about the degree of interdependence between the various model parameters. For a given extrinsic parameter, the normalized sensitivity coefficient was determined using Eq 12 [34]:

$$S_{\theta_{i}}=\frac{\theta_{i}}{E}\left(\frac{\partial E}{\partial \theta_{i}}\right)$$ (12)

where *E* is the sum of squared difference between experimental data (Figs 2–4) and the integrated bioenergetics model fit, and $\theta_{i}$ is the estimated value of *i*^th^ intrinsic parameter. $\frac{\partial E}{\partial \theta_{i}}$was approximated using the central difference method with 0.1% change in $\theta_{i}$.

The matrix of correlation coefficients between the model parameters was evaluated at the values in Table 2 that best fit the integrated model to the data (Figs 2–4). The correlation coefficient (*CC*_*ij*_) between the ith parameter and jth parameter was determined using Eq 13 [35]:

$$CC_{ij}=\frac{HH_{ij}}{\sqrt{HH_{ii}*HH_{jj}}}\;\;\;\;\text{for}i,\;j=1,\;\ldots ,\;np$$ (13)

where *np* is the number of model parameters, *HH* is the inverse of the product of the transpose of the Jacobian matrix and the Jacobian matrix evaluated at the values of the model parameters in Table 2 that best fit the integrated bioenergetics model to the data in Figs 2–4.

**Fig 5:**
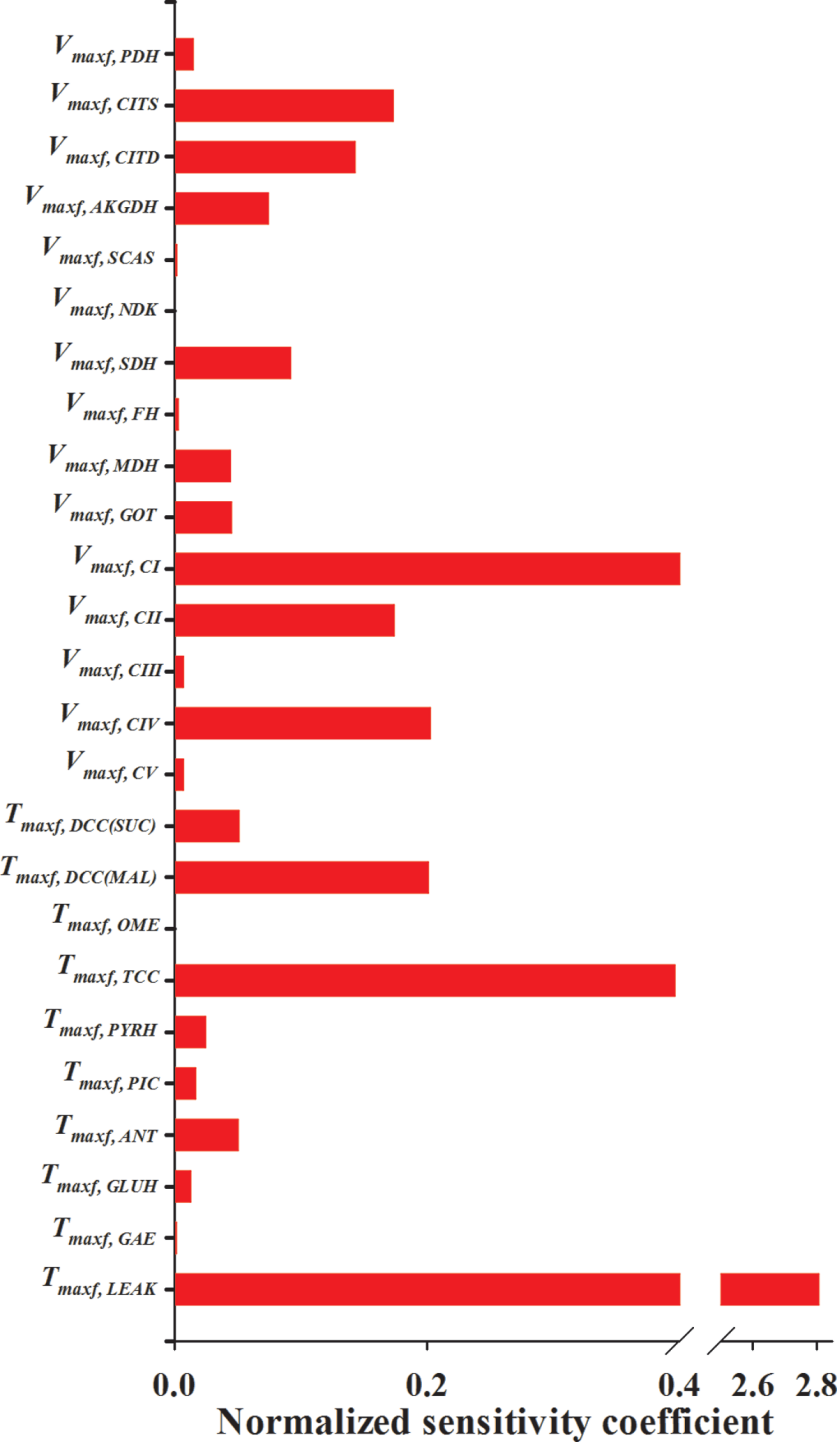
Normalized sensitivity coefficients of the integrated model extrinsic parameters. A parameter contribution to the model solution is proportional to its normalized sensitivity coefficient estimated using Eq 12.

**Fig 6:**
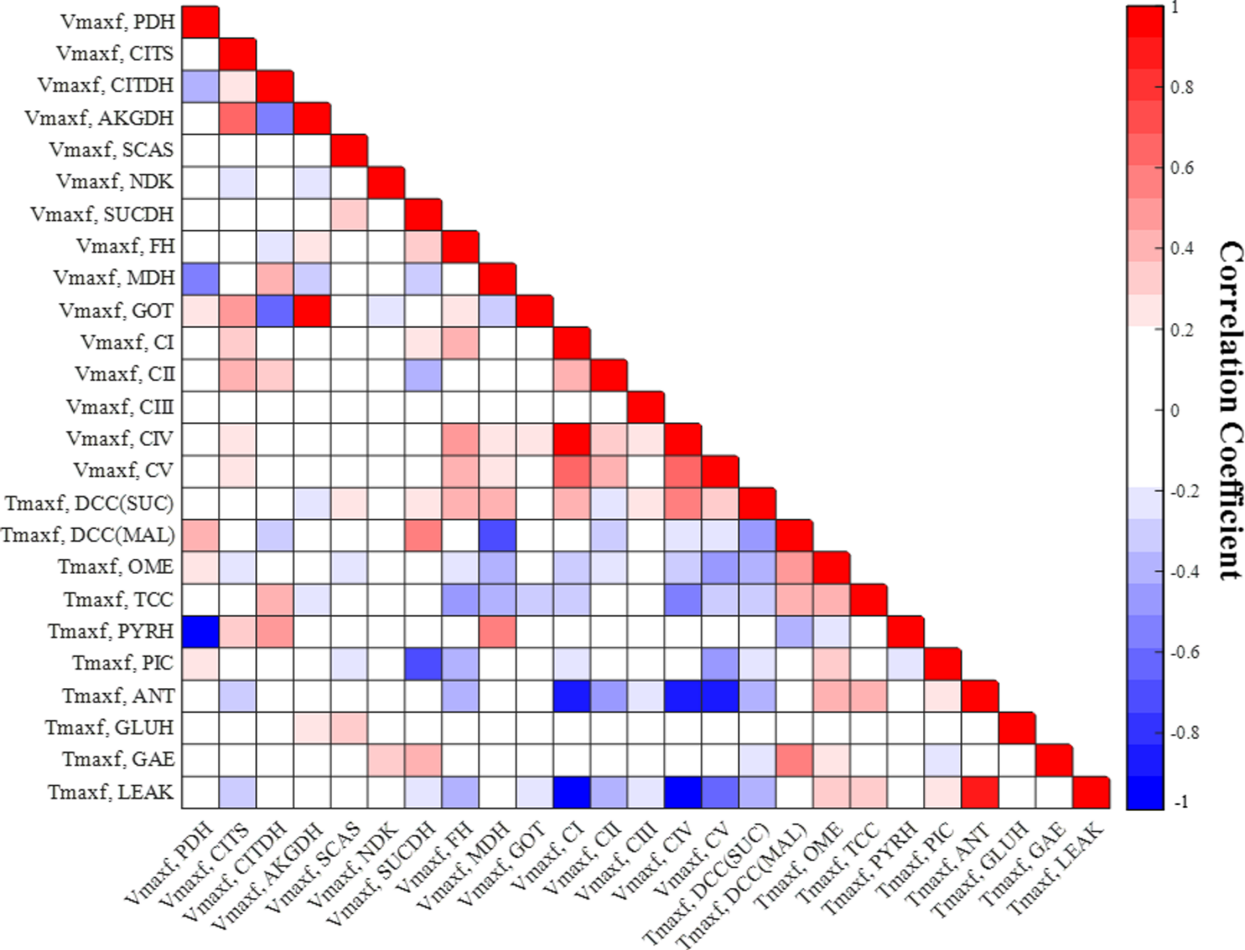
Matrix of correlation coefficients between the extrinsic parameters of the integrated model. Correlation coefficients range between -1 (perfect negative coefficient) and +1 (perfect positive coefficient) and are estimated using Eq 13. A small positive or negative correlation coefficient between two parameters suggests small interdependence between those parameters.

Figs 5 and 6 show the respective normalized sensitivity coefficients and matrix of correlation coefficients for the intrinsic parameters of the integrated model. For most of the intrinsic model parameters, the normalized sensitivity functions are relatively high, and the correlation coefficients are relatively low, consistent with a tight range of values for those parameters that provide a good fit to the data in Figs 2–4.

### Experimental and computational results and model validation

#### Oxygen consumption

**Fig 7:**
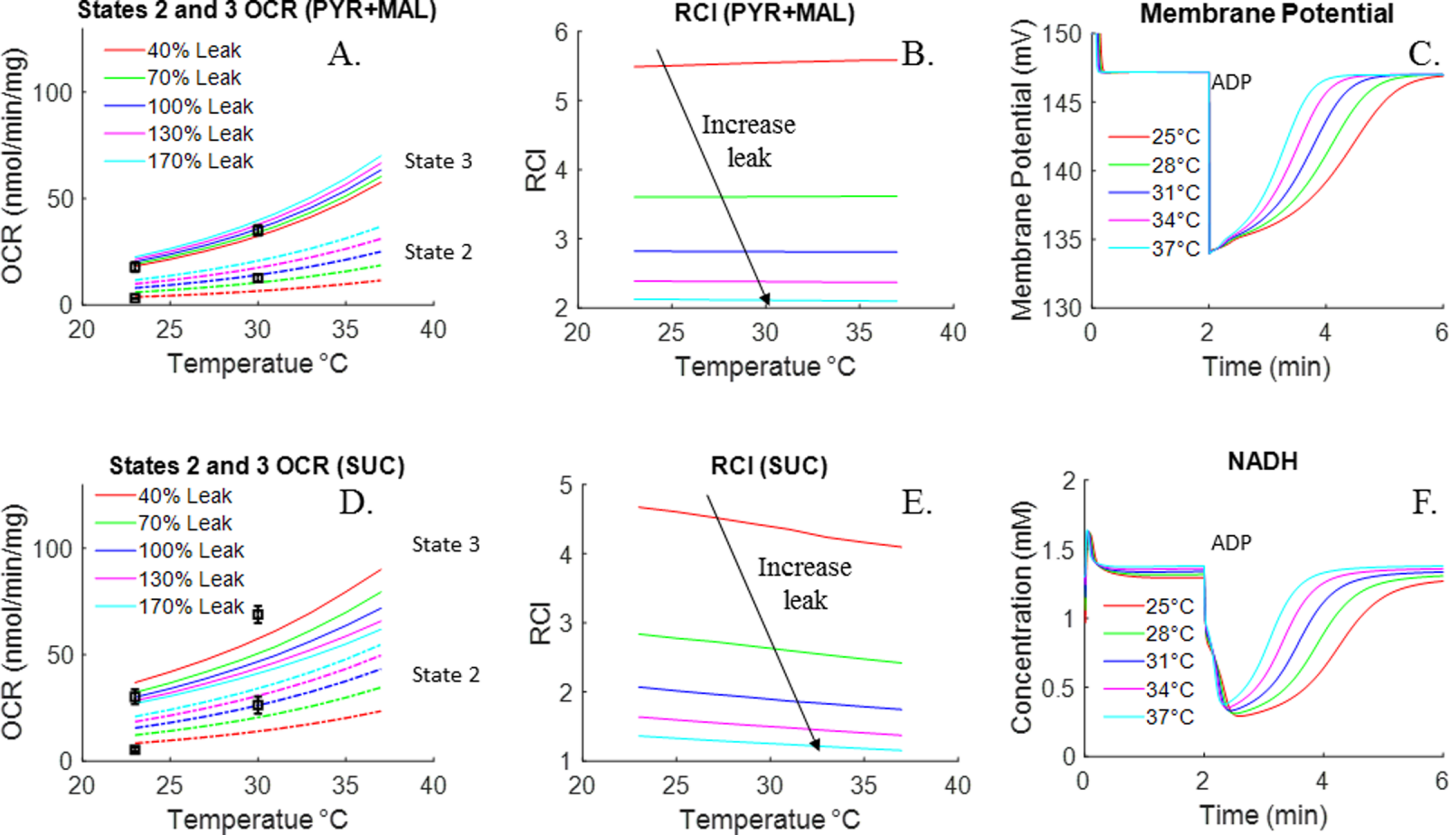
Effect of temperature and/or proton leak activity on mitochondrial respiration, membrane potential, and NADH concentration. Fig 7A (PYR+MAL) and Fig 7D (SUC) show integrated bioenergetics model simulation of the state 2 and 3 oxygen consumption rates (OCR) with different proton leak activities at different temperatures. Temperature was varied from 25 to 37°C and leak activities were varied from 40% to 170% of *T*_*maxf*, *LEAK*_ value in Table 2. Symbols are experimental data (mean ± SE) of Figs 3, 4 and 7B (PYR + MAL) and 7E (SUC) show corresponding simulated RCIs, which were calculated as the ratio of state OCR and state 2 OCR. Fig 7C and 7F show model predictions of membrane potential and NADH dynamic responses at different temperatures. For the model predictions, $V_{m}^{APP}$was set to 2.25 µl/mg mitochondria protein and $V_{e}^{APP}$was set to that for *V*_*e*_ = 1 ml.

Respirometry data measured at 23°C are shown in Fig 4. Averaged time-course respirometry data (mean ± SE) are shown in the presence of complex I substrates pyruvate + malate (Fig 4A, n = 4), complex I substrates malate + glutamate (Fig 4B, n = 1), and complex II substrate succinate (Fig 4C, n = 4). A summary of the oxygen consumption rates (OCR, nmol/min/mg) show higher states 2, 3 and 4 respiratory rates with complex II substrate (Fig 4F) as compare to those with complex I substrates (Fig 4D and 4E). Furthermore, the results show smaller respiratory rates, but higher respiratory control index (RCI, defined as the ratio of state 3 OCR to state 4 OCR) than those in Fig 3 (at 30°C) for pyruvate + malate and succinate. Fig 4 also shows fits of the integrated bioenergetics model to the measured oxygen consumption kinetic data (solid lines, upper panels) and oxygen consumption rates (red bars, lower panels) generated using the values of the model parameters in Table 2, except for the value of the *T*_*maxf*_ parameter descriptive of the proton leak process (*T*_*maxf*, *LEAK*_), which was set at 40% of the value in Table 2. This is consistent with a less “leaky” and more coupled isolated rat lung mitochondria for the data in Fig 4 as compared to the data in Figs 2, 3, 7A and 7D, which provide a summary of the states 2 and 3 oxygen consumption rates in Figs 3 and 4, show the expected dependency of those rates on buffer temperature.

#### Complex IV activity and integrity of mitochondrial outer membrane

Maximal complex IV activity was measured by adding TMPD (artificial electron donor for complex IV) along with ascorbate to the medium in the presence of antimycin A. The measured oxygen consumption rates (OCR) are shown in Table 3, which also shows OCR in the presence of exogenous cytochrome C and succinate. The results in Table 3 show no significant difference between the oxygen consumption rates measured under state 3 conditions in the presence of succinate and those measured in the presence of TMPD + ascorbate or TMPD + ascorbate + cytochrome C. These results are consistent with those reported by Fisher et al. [24, 27].

**Table 3:** Complex IV activity (nmol/min/mg).

Electron donor	OCR (nmol/min/mg) Current study (30°C)	OCR (nmol/min/mg) Fisher et al. [24, 27], 28°C
**TMPD+Ascorbate**	65 ± 12 (n = 8)	50 ± 13 (n = 4–7)
**TMPD+Ascorbate+CytoC**	73 ± 11 (n = 8)	NA
**SUC+ADP**	78± 11 (n = 8)	53 ± 9 (n = 4–7)

Values are mean ± SE. OCR, oxygen consumption rate; CytoC, cytrochrome c; TMPD, tetramethyl-p-phenylenediamine; NA, not available.

#### Mitochondrial membrane potential

**Fig 8:**
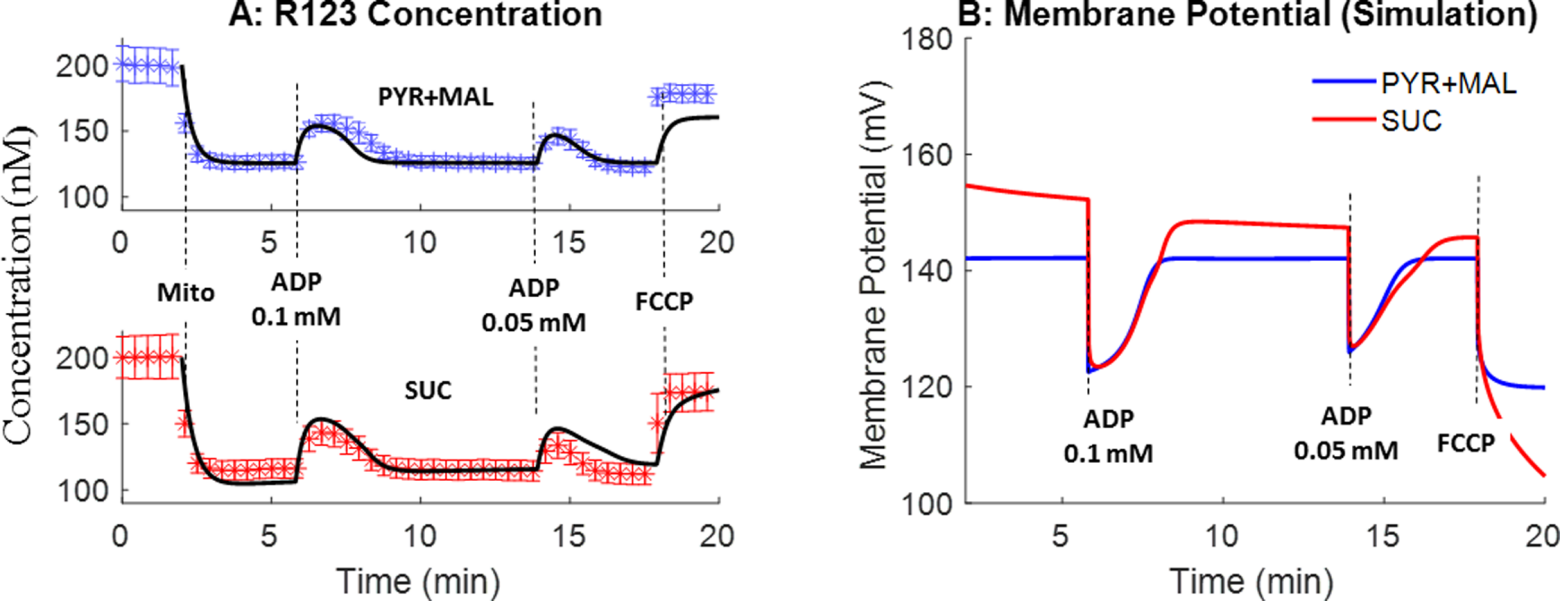
Model validation using membrane potential data. Experiments were performed under the same experimental condition as in Fig 4. R123 buffer concentrations are shown (symbols). Either pyruvate + malate (PYR+MAL, blue, n = 4) or succinate (SUC, red, n = 4) was used as the metabolic substrate. Values are mean ± SE. Black lines in Fig 8A are model predictions using the estimated values of the model parameters in Table 2. Fig 8B shows integrated model predictions of the mitochondrial membrane potential with either PYR + MAL or SUC as the metabolic substrates and with the values of the model parameters set to those in Table 2.

We quantified ADP-stimulated depolarization of ΔΨ_m_ in mitochondria isolated from rat lungs using R123 in the presence of complex I (pyruvate + malate) or compex II (succinate) substrates at 23°C. Fig 8A shows that in the presence of mitochondria and complex I substrates, the addition of ADP (state 3) stimulated a transient and reversible efflux of R123 from mitochondria, consistent with transient and reversible partial depolarization of ΔΨ_m_. The addition of 0.1 mM ADP resulted in a greater and longer membrane potential depolarization than the addition of 0.05 mM ADP. The addition of the mitochondrial uncoupler FCCP at the end of the protocol maximally depolarized ΔΨ_m_ resulting in the maximal efflux of R123 from mitochondria [20]. Higher membrane potential (thus lower R123 concentration in buffer) was obtained with succinate as a complex II substrate using the same protocol. Fig 8A also shows the simulated (model predicted) R123 dye concentration in buffer using the integrated bionergetics model and the pharmacokinetic model of rhodamine 123 mitocondrial uptake with the bioenergetics model parameter values set to those in Table 2 estimated from data in Figs 2–4. As shown in Fig 8A, model simulations are in good agreement with the measured experimental data. Fig 8B shows the corresponding mitochondrial membrane potentials predicted using the integrated bioenergetics model under the same conditions as in Fig 8A.

#### Model prediction of ACoA and CoA steady-state data and dynamic NADH redox status data

**Fig 9:**
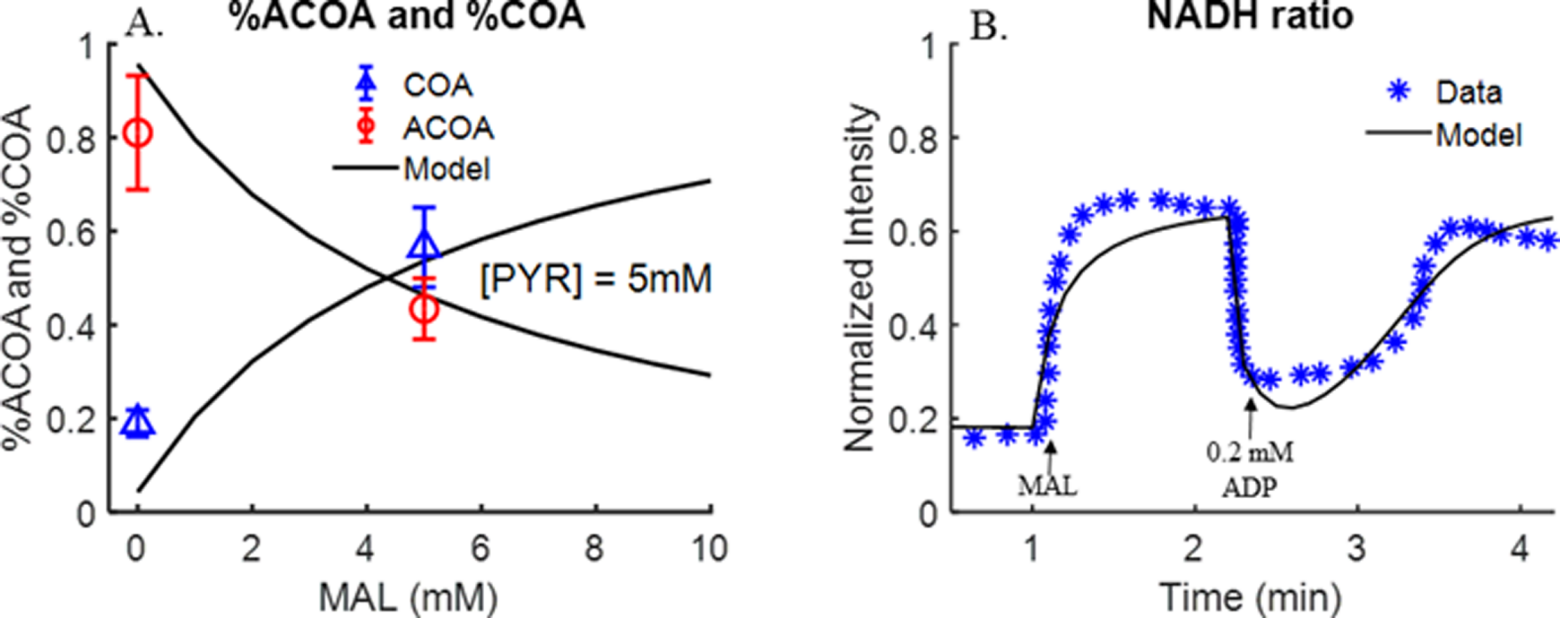
Model validation using ACoA and CoA steady state data (Evans et al. [27]) and dynamic NADH redox status data [24]. *Panel A*. Rat lung mitochondria were incubated with fixed pyruvate (PYR) concentration (5 mM), and without or with 5 mM malate (MAL). Mitochondria density = 2.5 mg/ml. Other experimental conditions are the same as in Fig 2. ACoA and CoA steady-state concentrations were measured at the end of 10-min incubation time. Symbols are experimental data and solid lines are integrated model predictions under the same experimental conditions with the values of the model parameters set to those in Table 2. *Panel B*. Fluorescence of pyridine nucleotides (NADH) in isolated rat lung mitochondria. Mitochondrial density was 2.5 mg/ml. 2.5 mM PYR, 2.5 mM MAL and 0.2 mM ADP were added to the buffer at 0 min, 1 min and 2.2 min, respectively. Symbols are experimental data and solid lines are integrated model predictions under the same experimental conditions with the values of the model parameters set to those in Table 2.

In addition to membrane potential data (Fig 8), we evaluated the ability of the integrated model to predict steady-state ACoA and CoA fractions at different malate concentrations [27], and kinetic NADH redox status data under different respiratory states [24]. Fig 9A and 9B show the ability of the integrated bioenergetics model to predict quit well both sets of data using the values of the model parameters in Table 2 estimated from the data in Figs 2–4.

## Discussion

We have developed and validated the first integrated mechanistic computational model of the bioenergetics and respiration of isolated lung mitochondria using new and previously published experimental data. The model provides a mechanistic and quantitative framework for 1) integrating experimental data from isolated lung mitochondria under diverse experimental conditions, and 2) assessing the impact of a change in one or more mitochondrial processes on overall lung mitochondrial bioenergetics. In addition, as described below, this integrated model provides important insights into the bioenergetics and respiration of isolated lung mitochondria and how they differ from those of mitochondria from other organs.

Similar computational models of mitochondrial bioenergetics and respiration have been developed for mitochondria isolated from other organs, including the heart and skeletal muscles [16–18, 21, 23]. Such models differ from the current integrated model in many aspects, in part because of differences in mitochondrial bioenergetics and energy demand of lung tissue as compared to those of the heart and skeletal muscle. It is well known that mitochondrial content, major metabolic substrates, and control mechanisms vary widely between different organs. For instance, β-oxidation of fatty acids has been identified as the primary source of energy for heart under physiological conditions accounting for 70% of ATP production [36–38], whereas glucose is the primary source of energy for lung tissue and accounts for 80–85% of the total lung tissue ATP production under physiological conditions [8]. Moreover, lung cells are mostly non-excitable, and thus are postulated to behave very differently from excitable cells such as cardiac and skeletal muscle cells [39]. For instance, the ATP demand for cardiac cells between resting state and maximal exercise state could increase by ~4 fold, while that for skeletal muscle cells could increase by as much as ~100 fold between resting state and maximal exercise state [40, 41]. Thus, key enzymes in existing computational models are modeled to be modulated by metabolic controllers, such as inorganic phosphate (Pi), ADP/ATP ratio, NADH/NAD^+^ ratio, etc. However, in lung mitochondria where the energy requirement is much more stable, such control mechanisms may not be as important. Furthermore, most existing computational models place emphasis on complex I substrate-dependent respiration [18, 23]. These differences were one of the reasons for the development and validation of an integrated model specific for the bioenergetics and respiration of isolated lung mitochondria.

Another reason for developing a comprehensive integrated model for bioenergetics and respiration of isolated lung mitochondria is the relatively complex flux equations for enzymes and transporters used in the existing heart and skeletal muscle models [16–18]. Those equations are not feasible for an isolated lung mitochondrial model due to the scarcity of experimental data needed to estimate the values of the large number of unknown parameters in the individual flux expressions and in the integrated mitochondrial model [27]. In the present study, a simplified general flux equation that accounts for apparent binding constants was developed and utilized for all enzymatic reactions and metabolite transporters. As such, the number of unknown model parameters was minimized for the individual flux expressions as well as for the integrated bioenergetics model. Yet, the model incorporated major pathways associated with lung mitochondrial bioenergetics, including metabolite transports and oxidations, TCA cycle reactions, electron transport chain (ETC) reactions, and oxidative phosphorylation.

### Estimation of unknown model parameters, and integrated model predictions

For intrinsic model parameters, such as binding constants (*K’*s), for major enzyme reactions and transport processes, their values were estimated using experimental data measured in isolated enzymes or fixed to published values, mostly from organs other than lungs due to lack of such data from lungs. The assumption is that for a given mitochondrial enzyme or transporter, intrinsic properties, such as *binding constants K’s*, are organ-independent [18].

With intrinsic model parameters known, a challenging aspect of the integrated bioenergetics model development was estimation of the values of the extrinsic parameters (Table 2). To that end, we relied on previously published experimental data from multiple studies in isolated rat lung mitochondria as well as new data that were obtained in the present study for further validation and corroboration of the model. As shown in Figs 2–4 and 8–9, the model is capable of fitting (Figs 2–4) and predicting (Figs 8–9) quite well all the data from isolated rat lung mitochondria with the same set of values for the extrinsic model parameters (Table 2).

A genetic algorithm (GA) was used to estimate the values of the extrinsic model parameters that best fit the kinetic data in Figs 2–4. Even though GA is a global parameter estimation algorithm that does not require initial estimates for the model parameters, good initial estimates can significantly reduce the computational time needed to identify optimal values of the model parameters. Good initial estimates can be obtained from boundary conditions such as pyruvate uptake rate, citrate production rate, and malate production rate.

For the TCA cycle experimental data measured by Evans et al. [27] and used for estimating the values of some of the model parameters, citrate formation was reported even when no exogenous pyruvate was added to the buffer medium (Fig 2C and 2F). In our integrated bioenergetics model, both pyruvate and malate are required to generate citrate. This resulted in a difference between model fits and experimental data when the pyruvate buffer concentration is zero. One possible explanation for this apparent inconsistency is that citrate measured at zero pyruvate buffer concentration, in the experiments by Evans et al., was from endogenous substrates, such as ACoA [27]. However, reported endogenous ACoA of ~1 nmol/mg mitochondria [27] is too small to account for the measured 40 nmol citrate. Since citrate formation was observed only after 4 min of incubation time (as shown in Fig 2C and 2F), it is more likely that the source of citrate was other metabolic pathways that are not included in this model such as amino acids from mitochondrial proteins [42]. Another potential source of medium pyruvate is the presence of some endogenous pyruvate in the isolated mitochondria.

### Identifiability and estimability of the extrinsic parameters of the integrated model

Fig 5 shows that proton leak extrinsic parameter *T*_*maxf*, *LEAK*_ has the largest normalized sensitivity coefficient among the 25 extrinsic model parameters (Table 2) of the integrated model. This could be because mitochondrial membrane leakiness not only affects oxygen consumption rate, RCI, and membrane potential, but also substrate consumption rate at state 2 since proton leak is the only pathway in this model for consumption of the energy provided by substrate oxidation under state 2 conditions (in the absence of ADP).

The intrinsic model parameters for glutamate-hydrogen or glutamate-hydroxyl antiporter (GLUH), nucleoside diphosphokinase (NDK), fumarate hydratase (FH), and α-ketoglutarate (2-oxoglutarate) malate exchanger (OME) reactions have the smallest normalized sensitivities coefficients (Fig 5). GLUH exists in rat liver mitochondria and heart mitochondria [43], but does not exist in rat brain mitochondria [43]. The existence of GLUH in rat lung mitochondria has not been confirmed, even though it was proposed that GLUH might be an important process for the replenishment of TCA cycle metabolites in the rat lung [44]. In the present study, the estimated GLUH activity and sensitivity are very low, indicating that GLUH is not required to fit the data in Figs 2–4 or to predict the data in Figs 8 and 9. On the other hand, glutamate oxaloacetate (GOT) is the major pathway for glutamate + malate-driven respiration. The GOT enzyme activity reported by Evans et al. [27, 44] (975 nmol/min/mg protein) is both necessary and sufficient to reproduce the glutamate + malate respirometry data in Fig 4B. Since the NDK and FH reactions are running at near equilibrium, their corresponding extrinsic parameters are not identifiable without additional data.

We also estimated the matrix of correlation coefficients between the extrinsic parameters (Fig 6) of the integrated bioenergetics model for the parameter values in Table 2. The extrinsic parameter pairs with the highest positive correlation are those for PDH/PYRH and DCC(SUC)/SDH reactions. The extrinsic parameter pair with the highest negative correlation is that for TCC/CITDH reactions. Extrinsic parameters for equilibrium reactions such as NDH and FH have virtually no correlation with the extrinsic parameters of other reactions.

### Membrane integrity of isolated mitochondria

Isolation of mitochondria from rat lung tissue is challenging due to the high lipid content and low mitochondria content [45]. For instance, cardiac myocyte mitochondria account for ~35% of cell volume, compared to 1–2% in pulmonary endothelial cells, which account for 50% of the cells in the lung [45]. Thus, a relatively high concentration of bovine serum albumin is required in the isolation buffer to bind free fatty acid and lipids. In addition, the integrity of lung mitochondrial membrane in the isolated mitochondria is highly dependent on the isolation protocol used. For the data from Evans et al. (Fig 3), the measured state 2 oxygen consumption rate was higher than that measured in the present study (Fig 4). This could be due to differences in the mitochondria isolation protocols used. Thus for the model fit to the data in Figs 2–4, the value of the leak parameter (*T*_*maxf*, *LEAK*_, which accounts for state 2 respiratory rate) was adjusted to account for differences in the measured state 2 respiratory rate.

Simulations in Fig 7 show the effect of temperature and proton leak activity (*T*_*maxf*, *LEAK*_) on mitochondrial oxygen consumption. With pyruvate + malate as metabolic substrates, both state 2 and state 3 oxygen consumption rates increase (Fig 7A), although the % increase in state 2 appears to be larger than that for state 3. As a result, there is an inverse relationship between proton leak activity and RCI (Fig 7B). In contrast, with succinate as the metabolic substrate, the state 3 oxygen consumption rate decreases as proton leak activity increases (Fig 7E). Therefore, membrane leakiness has a higher impact on RCI when using succinate as substrate.

By altering the proton leak activity (*T*_*maxf*, *LEAK*_), the integrated model was capable of simulating a wide range of mitochondrial experimental data performed under different experimental conditions (temperature, mitochondria density, pH, etc.) with the same set of values for the other model parameters (adjusted for temperature and pH effects). Fig 7C and 7F show the effect of temperature on dynamic responses of membrane potential and NADH redox states at state 3. Fig 7C also shows that ADP is consumed faster at higher temperature. As a result, the length of state 3 decreases as temperature increases.

### Apparent functionally incomplete TCA cycle in isolated lung mitochondria

Isolated rat lung mitochondria and heart mitochondria show different behavior under similar experimental conditions. For instance, isolated rat lung mitochondria with pyruvate + malate as respiratory substrates produce significantly higher citrate than isolated cardiac mitochondria [26]. For lung mitochondria with pyruvate + malate as metabolic substrates, citrate production rate accounts for ~80% of the pyruvate and malate uptake rate. On the other hand, for heart mitochondria, the consumed pyruvate and malate rate is approximately equal to amounts of citrate, α-ketoglutarate, succinate, and fumarate production rates [26].

**Fig 10:**
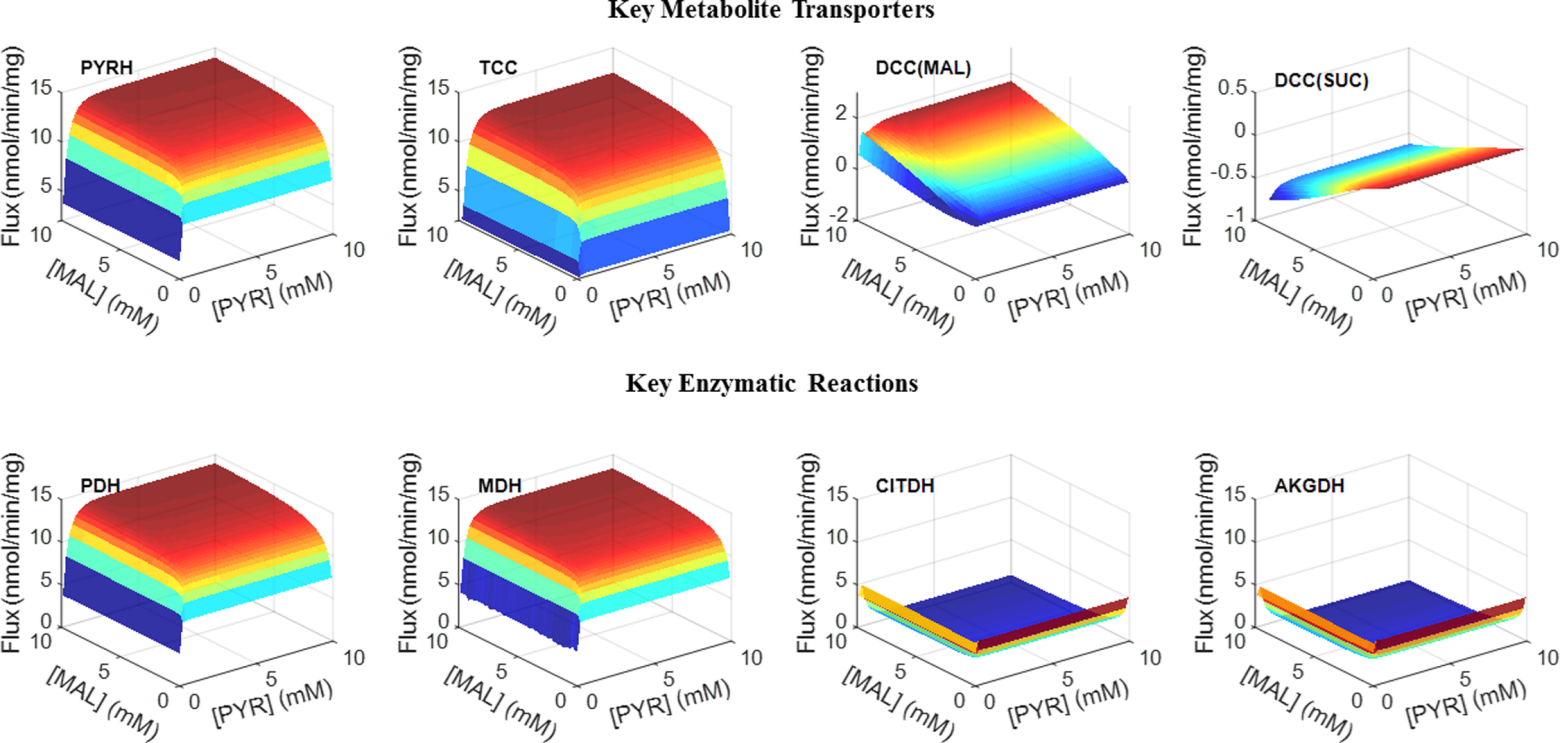
Model predicted substrate-dependent responses of the steady state reaction and transport fluxes. 3-Dimensional surface plots of key metabolic fluxes at steady-state as a function of buffer pyruvate (PYR) and malate (MAL) concentrations ranging from 0.5 mM to 10 mM obtained at the end of a 10-minute incubation period in isolated lung mitochondria. Simulations were performed under the same experimental conditions as in Figs 2 and 3 with the values of the extrinsic model parameters set to those in Table 2.

The large citrate production rate in isolated rat lung mitochondria is consistent with an apparent functionally incomplete TCA cycle since most of the consumed carbon from pyruvate and malate is converted to citrate and released into the extra-mitochondrial buffer medium. Therefore, not enough substrate is left for the reaction to proceed beyond citrate in the TCA cycle. This might also be due to high NADH significantly inhibiting the CITDH reaction under physiological conditions, based on isolated enzyme experiments, as shown in Fig 7A in S1 Supporting Information (*K*_NADH_ = 4.7 μM). Integrated bioenergetics model predicted simulations (Fig 10) also show that the reaction fluxes of CITDH and AKGDH are very low compared with those for PYRDH and MALDH.

Additional evidence of the apparent functionally incomplete TCA cycle in isolated rat lung mitochondria is the relatively high pyruvate/oxygen ratio since pyruvate consumption rate (10 nmol/min/mg mitochondria protein, as shown in Fig 2) is comparable to oxygen consumption rate (12 nmol/min/mg mitochondria protein, as shown in Fig 3) under the same experimental conditions with pyruvate + malate as metabolic substrates. If all the TCA cycle reactions are active, one would expect 4 NADH to be generated from each pyruvate (PYRDH, CITDH, MALDH and AKGDH), and 8 electrons to transfer to complex IV and reduce two molecules of oxygen. However, the data from Evans et al. (Figs 2 and 3) show that only 1.2 oxygen molecules are consumed for each pyruvate molecule. The high pyruvate/oxygen ratio in experiments suggests that only around 2.4 molecules of NADH are produced for each pyruvate consumed, consistent with only two of the four dehydrogenases being functional.

The observed large citrate production and the release to extra-mitochondrial buffer in isolated lung mitochondria with complex I substrate (pyruvate + malate) could be a means to ensure the availability of enough lipid synthesis for lung surfactant since citrate is an important source of acetyl units for ATP citrate lyase, an important step in fatty acid biosynthesis. The lung is an active organ that produces fatty acid to maintain lung surfactant balance, which is composed of approximately 90% lipids [46].

It is well known that calcium ions (Ca^2+^) have a stimulating effect on key dehydrogenase enzymes in mitochondria [41]. Considering that not all of the dehydrogenase enzymes are active in isolated mitochondrial experiments (Fig 10), the effect of Ca^2+^ control on the TCA cycle and bioenergetics might be underestimated in isolated mitochondrial experiments. For example, Vinnakota et al. [41] showed that physiological and supra-physiological Ca^2+^ levels have no or only modest stimulatory effect on cardiac mitochondrial oxidative phosphorylation, respectively, in isolated mitochondrial respiring on complex I substrates (pyruvate + malate), which is insufficient to explain the Ca^2+^ stimulating effect on oxidative phosphorylation *in vivo* in the heart. The apparent functionally incomplete TCA cycle in isolated lung mitochondria due to excess citrate production and release to buffer (Figs 2 and 10) could provide an explanation for why the stimulating effect of Ca^2+^ on mitochondrial bioenergetics and respiration with pyruvate + malate as respiratory substrate is less evident in isolated cardiac mitochondria than *in vivo*. In addition, significant NADH inhibition of the CITDH reaction at physiological conditions could be another mechanism behind the non-stimulating effects of Ca^2+^ on mitochondrial bioenergetics and respiration in isolated cardiac mitochondria with pyruvate + malate as respiratory substrates.

### Rate-limiting steps in rat lung mitochondrial electron transport chain (ETC)

In the present study, experimental data and model sensitivity analysis reveal key information regarding the limiting step(s) in the rat lung mitochondrial ETC. Consistent with previously reported data [24, 27], states 2 and 3 oxygen consumption rates in the presence of succinate were higher than those measured in the presence of pyruvate + malate.

In isolated heart mitochondria, simultaneous addition of complex I and complex II substrates increased state 3 oxygen consumption rate by up to 2-fold relative to that following the addition of complex I or complex II substrates alone [47]. In contrast to heart and liver mitochondria, our data show that convergent electron flow from both complex I substrates and complex II substrates is not additive (e.g. oxygen consumption rate with pyruvate + malate + succinate did not increase relative to that with succinate alone), indicating that in isolated rat lung mitochondria the rate limiting step(s) for the flow of electrons in the ETC is downstream from complexes I and II. Therefore, complexes III, IV, V and/or ANT could be the potential rate limiting step.

At the end of each isolated rat lung mitochondrial experiment in Fig 4, the uncoupler FCCP was added to the surrounding buffer to increase proton leak from the inter-membrane space to the mitochondria matrix. In agreement with a previous rat lung mitochondria study [24], our data (Fig 4 and Table 3) show that the oxygen consumption rate at uncoupled state 3 did not increase relative to that of ADP-stimulated state 3, suggesting that there is no excess ETC capacity over and above that for oxidative phosphorylation. Thus, electron flow under state 3 respiration is not limited by mitochondrial complex V or ANT. It is more likely that complex III and/or complex IV are the rate limiting steps in rat lung mitochondria.

Integrated model sensitivity analysis can reveal important information regarding rate limiting steps in mitochondrial ETC. A high sensitivity coefficient for a given parameter indicates that the model output is sensitive to the enzyme whose activity is described by that parameter. Therefore, an enzyme with a relatively high sensitivity coefficient is more likely to be the rate limiting step than an enzyme with a low sensitivity coefficient. Sensitivity analysis results (Fig 5) show that mitochondrial complex I and complex IV have much higher normalized sensitivity coefficients than complex III. One possible explanation is that with complex I substrates, there is excess electron transport capacity in ETC, and that mitochondrial electron flow is limited by complex I activity. However, when complex II substrate is present, electron flow from complexes I and II exceeds the maximum capacity of complex IV, and thus complex IV becomes the rate limiting step.

To prove that mitochondrial complex IV is the rate limiting step, maximum complex IV activity was measured using the complex IV substrate TMPD + ascorbate as direct electron donors. Results are compared with oxygen consumption rate with succinate as the metabolic substrate. As shown in Table 3, ADP-stimulated maximum respiration rate (state 3) in the presence of succinate was not different from that in the presence of TMPD + ascorbate. Similar results were reported by Fisher et al. [24, 27] (Table 3). These results suggest that maximum complex IV activity is reached when succinate is used as the metabolic substrate, and that additional oxygen consumption cannot be stimulated by the addition of an uncoupler or complex I substrates to the surrounding medium.

Release of cytochrome c due to the relatively leaky mitochondrial outer-membrane could be a reason for the apparent low complex IV activity. If so, the addition of exogenous cytochrome c to the medium should markedly stimulate oxygen consumption. As shown in Table 3, adding exogenous cytochrome c had a small (12.5%) and insignificant effect (*paired t-test*, *p = 0*.*11*) on the measured rate in the absence of exogenous cytochrome c in the medium. Considering that a ≤10% increase of the cytochrome c stimulated oxygen consumption can be regarded as a sign of intact outer-membrane in heart and muscle mitochondria [48], a 12.5% increase in oxygen consumption may suggest a slightly “leaky” mitochondrial outer-membrane.

### Pharmacokinetic model for R123 mitochondrial uptake from medium

A novel feature of the present study is the coupling of a pharmacokinetic model for R123 mitochondrial uptake to the integrated bioenergetics model to predict the measured R123 medium concentration and quantify mitochondrial membrane potential (Fig 8). R123 fluorescence intensity increases linearly with dye concentration at low concentrations (<1 µM) in the buffer [20]. Thus, we converted the R123 fluorescence intensity to R123 concentration to compare with model simulated R123 concentration in the buffer. Integrated model simulations in Fig 8 show good agreement with experimental data. In addition, integrated model predicted lung mitochondrial membrane potential (~140 mV) is close to the value (133 ± 4 (SE) mV) measured by Gan et al. in cultued bovine pulmonary artery endothelial cells [33] and to the value (129 ± 4 (SE) mV) meaured by Bongard et al. in cultured rat pulmonary microvascular endothelial cells [9].

### Limitations and future directions

Metal ions such as calcium (Ca^2+^) and magnesium (Mg^2+^) ions play an important role in rat lung mitochondrial bioenergetics. In this integrated bioenergetics model, all the metal ion concentrations are assumed constant. Even though it is known that dehydrogenases are activated by Ca^2+^ at nM level (~100–300 nM), Fisher et al. reported that mitochondrial oxygen consumption rates are inhibited as Ca^2+^ concentration increases to µM-mM ranges [24]. However, the detailed mechanism for this biphasic effect is not known. Therefore, additional studies and data from isolated rat lung mitochondria are required to incorporate metal ions into the current model.

Another limitation of this model is that it does not differentiate between the forty different cell types in the lung. Endothelial cells, which account for ~50% of all lung cells, are not rich in mitochondrial [33]. However, other lung cell types including contractile (smooth muscle cells), phagocytic (alveolar macrophages) and epithelial cells are rich in mitochondria. Thus, the model and the estimated values of model parameters should be interpreted as a mechanistic description of the average mitochondrial bioenergetics of all lung cells. Although the question regarding the mitochondrial bioenergetics and respiration of specific lung cell types is important, understanding the bioenergetics/respiration of mitochondria isolated from lung tissue and alteration in those bioenergetics/respiration is important and has functional implications regardless of the lung cell types involved.

The proposed integrated bioenergetics model is an important step towards the development and validation of a comprehensive thermodynamically-constrained computational model of isolated perfused rat lung tissue bioenergetics that includes cytosolic processes. Such a model will be used to evaluate the impact of ALI- or ARDS-induced changes in one or more mitochondrial or cytosolic processes on overall lung tissue bioenergetics, and for assessing the impact of targeting specific processes for mitigating the impact of ALI and ARDS on lung tissue bioenergetics.

## Supporting information

S1 Supporting Information Reactions and transport processes included in the integrated bioenergetics model along with model governing mass balance equations. This file consists of four parts. Part A lists the reaction and transport processes in the mitochondrial bioenergetics model. Part B provides a description of the derivation of the generalized metabolic reaction and transport flux equations. Part C lists the flux expressions for specific metabolic reactions and transport processes. Part D lists the governing mass balance equations for the mitochondrial bioenergetics model.
(DOCX)
