# Supplementary material for: Integrated computational model of the bioenergetics of isolated lung mitochondria
Source: PLoS One. 2018 Jun 11;13(6):e0197921. doi: 10.1371/journal.pone.0197921 (PMC5995348; doi:10.1371/journal.pone.0197921)
Supplement: S1 Supporting Information — This file consists of four parts. Part A lists the reaction and transport processes in the mitochondrial bioenergetics model. Part B provides a description of the derivation of the generalized metabolic reaction and transport flux equations. Part C lists the flux expressions for specific metabolic reactions and transport processes. Part D lists the governing mass balance equations for the mitochondrial bioenergetics model. (DOCX) [file pone.0197921.s001.docx]

# Supporting information

**Introduction**

This supporting information file consists of four parts. *Part A* lists the reaction and transport processes in the integrated model of the bioenergetics of isolated lung mitochondria. In what follows, we refer to this model as the integrated bioenergetics model. *Part B* provides a description of the derivation of the generalized metabolic reaction and transport flux equations. *Part C* lists the flux expressions for specific metabolic reaction and transport processes. *Part D* lists the governing mass balance equations of the integrated bioenergetics model.

| Terms | Description |
| --- | --- |
| Subscript m | Mitochondria matrix region |
| Subscript e | Extra-mitochondria (buffer) region |
| Subscript i | Inter-membrane space (IMS) region |
| Species | Description |
| ADP | Adenosine diphosphate |
| ATP | Adenosine triphosphate |
| ACoA | Acetyl-coenzyme A |
| ASP | Aspartate |
| AKG | α-ketoglutarate (2-oxoglutarate) |
| CoA | Coenzyme A |
| CIT | Citrate |
| CytCr | Reduced form of cytochrome c |
| CytCo | Oxidized form of cytochrome c |
| FUM | Fumarate |
| FAD | Oxidized form of flavin adenine dinucleotide |
| FADH_2_ | Reduced form of flavin adenine dinucleotide |
| GDP | Guanidine diphosphate |
| GTP | Guanidine triphosphate |
| GLU | Glutamate |
| MAL | Malate |
| OXA | Oxaloacetate |
| PYR | Pyruvate |
| Pi | Inorganic phosphate |
| SUC | Succinate |
| SCoA | Succinyl-coenzyme A |
| UQH_2_ | Reduced form of ubiquinone |
| UQ | Oxidized form of ubiquinone |

**Table A1. Glossary**

| Reactions | Description |
| --- | --- |
| AKGDH | α-ketoglutarate dehydrogenase |
| CITS | Citrate synthase |
| CITDH | Lumped reaction of aconitase and isocitrate dehydrogenase |
| CI | Complex I |
| CII | Complex II |
| CIII | Complex III |
| CIV | Complex IV |
| CV | Complex V |
| FH | Fumarate hydratase |
| GOT | Glutamate oxaloacetate |
| MDH | Malate dehydrogenase |
| NDK | Nucleoside diphosphokinase |
| PDH | Pyruvate dehydrogenase |
| SCAS | Succinyl-coenzyme A synthetase |
| SDH | Succinate dehydrogenase |
| Transporters | Description |
| ANT | Adenine nucleotide translocase |
| DCC | Dicarboxylate carrier |
| GAE | Glutamate-aspartate exchanger |
| GLUH | Glutamate-hydrogen co-transporter |
| HLEAK | Passive proton leak from inter-membrane space to mitochondria matrix |
| OME | α-ketoglutarate (2-oxoglutarate) malate exchanger |
| PIC | Inorganic phosphate carrier |
| PYRH | Pyruvate-hydrogen co-transporter |
| TCC | Tricarboxylate carrier |

**Part A: Reaction and transport processes in the integrated bioenergetics model**

All the enzymatic reactions considered in this integrated bioenergetics model are listed in Table A2, and all the transport processes are listed in Table A3.

Table A2. Enzymatic reactions in the mitochondria bioenergetics model

| Reaction number | Enzyme name | Enzyme reaction |
| --- | --- | --- |
| 1 | PDH | PYR_m_+CoA_m_+NAD_m_ ⇌ ACoA_m_+CO_2_+NADH_m_+H_m_ |
| 2 | CITS | ACoA_m_+OXA_m_ ⇌ CIT_m_+CoA_m_+ 2H_m_ |
| 3 | CITDH | CIT_m_+NAD_m_ ⇌ AKG_m_+NADH_m_+CO_2_ |
| 4 | AKGDH | AKG_m_+CoA_m_+NAD_m_ ⇌ SCoA_m_+NADH_m_+CO_2_ |
| 5 | SCAS | SCoA_m_+GDP_m_+Pi_m_ ⇌ SUC_m_+GTP_m_+CoA_m_+H_m_ |
| 6 | NDK | GTP_m_+ADP_m_ ⇌ GDP_m_+ATP_m_ |
| 7 | SDH | SUC_m_+FAD_m_⇌ FUM_m_+FADH_2m_ |
| 8 | FH | FUM_m_+H_2_O= MAL_m_ |
| 9 | MDH | MAL_m_+NAD_m_ ⇌ OXA_m_+NADH_m_+ H_m_ |
| 10 | GOT | ASP_m_+AKG_m_ ⇌ GLU_m_+OXA_m_ |
| 11 | CI | NADH_m_+UQ_m_+H_m_ ⇌ NAD_m_+UQH_2m_+ 4∆H |
| 12 | CII | FADH_2_+UQ_m_ ⇌ FAD_m_+UQH_2m_ |
| 13 | CIII | UQH_2m_+2CytCo ⇌ UQ_m_+2CytCr+ 2∆H+ 2$H_{i}$ |
| 14 | CIV | 2CytCr+0.5O_2_+2H_m_ ⇌ 2CytCo+H_2_O+4∆H |
| 15 | CV | ADP_m_+Pi_m_+H_m_⇌ ATP_m_+3∆H |

Table A3. Metabolite transporters in the mitochondrial bioenergetics model

| Transporter number | Transporter name | Transporter reaction |
| --- | --- | --- |
| 1 | PYRH | PYR_e_ + H_e_ ⇌ PYR_m_ + H_m_ |
| 2 | GLUH | GLU_e_+ H_e_ ⇌ GLU_m_+ H_m_ |
| 3 | DCC (MAL) | MAL_e_+Pi_m_ ⇌MAL_m_+Pi_e_ |
| 4 | DCC (SUC) | SUC_e_+Pi_m_ ⇌ SUC_m_+Pi_e_ |
| 5 | TCC | MAL_e_+ CIT_m_ ⇌ MAL_m_+ CIT_e_ |
| 6 | OME | MAL_e_+AKG_m_ ⇌ MAL_m_+AKG_e_ |
| 7 | GAE | ASP_e_+ HGLU_m_ ⇌ ASP_m_ + HGLU_e_ |
| 8 | ANT | ADP_e_ + ATP_m_ ⇌ ADP_m_ + ATP_e_ |
| 9 | PIC | Pi_e_ + H_e_ ⇌ Pi_m_ + H_m_ |
| 10 | Hleak | H_i_ ⇌ H_m_ |

## Part B: Derivation of the generalized metabolic reaction and transport flux equations:

### One-substrate one-product enzymatic reactions: $S\leftrightarrow^{E} P$

The general scheme for a reversible one-substrate one-product enzymatic reaction [1] is shown in Fig A1,

Fig A1. General scheme for one substrate and one product enzymatic reactions

where *E* is the enzyme, *S* is the substrate, *P* is the product, and *ESP* is a hypothetical enzyme-substrate-product complex that binds both substrate and product, *K_S_* is the dissociation constant of *ES* complex, and *K_P_* is the dissociation constant of *EP*. For simplicity and to reduce the number of unknown parameters, two assumptions were made to derive the general flux equations. First, we assumed rapid equilibrium conditions, i.e., all the enzymatic complex dissociation reactions are considered extremely rapid compared to the interconversion of *ES* and *EP*, which is the only rate limiting reaction step. Second, we assumed that the binding of *S* does not affect the binding of *P.*

Under the above assumptions, the flux equation for the reversible one-substrate one-product enzymatic reaction is given by

$\frac{J}{E_{t}}=\frac{k_{p}\left[ES\right]-k_{-p}\left[EP\right]}{E_{t}}=\frac{k_{p}\left[ES\right]-k_{-p}\left[EP\right]}{\left[E\right]+\left[ES\right]+\left[EP\right]+\left[ESP\right]}$ (A1)

$J=\frac{V_{\max f}\frac{\left[S\right]}{K_{S}}-V_{\max r}\frac{\left[P\right]}{K_{P}}}{1+\frac{\left[S\right]}{K_{S}}+\frac{\left[P\right]}{K_{P}}+\frac{\left[S\right]}{K_{S}}\frac{\left[P\right]}{K_{P}}}=\frac{\left(V_{\max f}\frac{\left[S\right]}{K_{S}}-V_{\max r}\frac{\left[P\right]}{K_{P}}\right)}{\left(1+\frac{\left[S\right]}{K_{S}}\right)\left(1+\frac{\left[P\right]}{K_{P}}\right)}$ (A2)

where *E_t_* is the total concentration of the enzyme, and *V_maxf_* = *k_p_.E_t_* and *V_maxr_* = *k_-p_.E_t_* are the maximum forward and reverse reaction velocities, respectively. The denominator of Eq A1 reflects all four species in this system, namely *E*, *ES*, *EP*, and *ESP*. However, under the assumption that both the substrate and product do not bind to the enzyme at the same time, the *ESP* complex does not form, and hence [*ESP*] = 0. Under this assumption, the flux equation for the reversible one-substrate one-product enzymatic reaction can be reduced to:

$J=\frac{V_{\max f}\frac{\left[S\right]}{K_{S}}-V_{\max r}\frac{\left[P\right]}{K_{P}}}{1+\frac{\left[S\right]}{K_{S}}+\frac{\left[P\right]}{K_{P}}}$ (A3)

We note here that the flux expression (A2) or (A3) for a reversible one-substrate one-product enzymatic reaction contains two intrinsic model parameters (enzymatic complex dissociation constants *K_S_* and *K_P_*; also referred to as binding constants), and two extrinsic model parameters (forward and reverse reaction maximal velocities *V_maxf_* and *V_maxr_*). The intrinsic parameters are assumed to be independent of tissue source of enzymatic reaction, while the extrinsic model parameters are assumed to be dependent on tissue source of enzymatic reaction. Specifically, the extrinsic model parameters are assumed to be dependent on the activity of the enzyme in a particular tissue, which is dependent on several cellular factors, such as enzyme expression, concentration, post-translational modifications, physiological state of the tissue, etc.

### Two-substrates two-products enzymatic reactions: $S_{1}+S_{2}\leftrightarrow^{E} P_{1}+P_{2}$

Fig A2. General scheme for two substrates two products enzymatic reactions

The general scheme (a random rapid-equilibrium binding scheme) for a reversible two-substrate two-product enzymatic reaction is shown in Fig A2. With the similar assumptions, as described for a reversible one-substrate one-product enzymatic reaction, the flux equation for a reversible two-substrate two-product enzymatic reaction can be derived as:

$J =\frac{V_{\max f}\frac{\left[S_{1}\right]\left[S_{2}\right]}{K_{S1}K_{S2}}-V_{\max r}\frac{\left[P_{1}\right]\left[P_{2}\right]}{K_{P1}K_{P2}}}{\begin{matrix} 1+\frac{\left[S_{1}\right]}{K_{S1}}+\frac{\left[S_{2}\right]}{K_{S2}}+\frac{\left[P_{1}\right]}{K_{P1}}+\frac{\left[P_{2}\right]}{K_{P2}}+\frac{\left[S_{1}\right]\left[P_{2}\right]}{K_{S1}K_{P2}}+\frac{\left[S_{1}\right]\left[S_{2}\right]}{K_{S1}K_{S2}}+\frac{\left[P_{1}\right]\left[P_{2}\right]}{K_{P1}K_{P2}}+\frac{\left[S_{2}\right]\left[P_{1}\right]}{K_{S2}K_{P1}}+\frac{\left[S_{1}\right]\left[P_{1}\right]}{K_{S1}K_{P1}}+\frac{\left[S_{2}\right]\left[P_{2}\right]}{K_{S2}K_{P2}} \\ +\frac{\left[S_{1}\right]\left[S_{2}\right]\left[P_{1}\right]}{K_{S1}K_{S2}K_{P1}}+\frac{\left[S_{1}\right]\left[S_{2}\right]\left[P_{2}\right]}{K_{S1}K_{S2}K_{P2}}+\frac{\left[S_{1}\right]\left[P_{1}\right]\left[P_{2}\right]}{K_{S1}K_{P1}K_{P2}}+\frac{\left[S_{2}\right]\left[P_{1}\right]\left[P_{2}\right]}{K_{S2}K_{P1}K_{P2}}+\frac{\left[S_{1}\right]\left[S_{2}\right]\left[P_{1}\right]\left[P_{2}\right]}{K_{S1}K_{S2}K_{P1}K_{P2}} \end{matrix}}$ (A4)

$J=\frac{\left(V_{\max f}\frac{\left[S_{1}\right]\left[S_{2}\right]}{K_{S1}K_{S2}}-V_{\max r}\frac{\left[P_{1}\right]\left[P_{2}\right]}{K_{P1}K_{P2}}\right)}{\left(1+\frac{\left[S_{1}\right]}{K_{S1}}\right)\left(1+\frac{\left[S_{2}\right]}{K_{S2}}\right)\left(1+\frac{\left[P_{1}\right]}{K_{P1}}\right)\left(1+\frac{\left[P_{2}\right]}{K_{P2}}\right)}$ (A5)

Considering that $S_{1}$ and $P_{1}$ share the same binding site on the enzyme, and $S_{2}$ and $P_{2}$ share a different binding site on the enzyme, as usually is the case for reaction cofactors (such as NADH and NAD^+^, ATP and ADP, GTP and GDP, etc.), the flux equation can be modified to exclude the contributions of ${[S}_{1}][P_{1}]$ or [$S_{2}][P_{2}]$ product terms:

$J=\frac{\left(V_{\max f}\frac{\left[S_{1}\right]\left[S_{2}\right]}{K_{S1}K_{S2}}-V_{\max r}\frac{\left[P_{1}\right]\left[P_{2}\right]}{K_{P1}K_{P2}}\right)}{\left(1+\frac{\left[S_{1}\right]}{K_{S1}}+\frac{\left[P_{1}\right]}{K_{P1}}\right)\left(1+\frac{\left[S_{2}\right]}{K_{S2}}+\frac{\left[P_{2}\right]}{K_{P2}}\right)}$ (A6)

The flux expression (A5) or (A6) for a reversible two-substrate two-product enzymatic reaction contains four intrinsic model parameters (binding constants *K_S1_*, *K_S2_, K_P1_* and *K_P2_*) and two extrinsic model parameters (forward and reverse reaction maximal velocities *V_maxf_* and *V_maxr_*), having the similar tissue-independent and tissue-dependent characteristics, as described above for the one-substrate one-product enzymatic reaction.

### Multi-substrates multi-products enzymatic reactions:

For more complex enzymatic reactions that contain multiple substrates and multiple products, we write the generalized reaction as:

$\sum_{i=1}^{N_{s}} \alpha_{i}S_{i}\leftrightarrow\sum_{j=1}^{N_{P}} \beta_{j}P_{j}$ (A7)

where *S_i_* is i^th^ substrate, *P_j_* is j^th^ product, *N_S_* and *N_P_* are number of substrates and products in the reaction, respectively, and *α_i_* and *β_j_* are the corresponding stoichiometric coefficients. The general form of the reaction flux *J* is given by

$J=\frac{V_{maxf}\frac{\prod_{i=1}^{N_{s}} \left[S_{i}\right]^{\alpha_{i}}}{\prod_{i=1}^{N_{s}} K_{S_{i}}^{\alpha_{i}}}-V_{maxr}\frac{\prod_{j=1}^{N_{p}} \left[P_{j}\right]^{\beta_{j}}}{\prod_{j=1}^{N_{p}} K_{P_{j}}^{\beta_{j}}}}{\prod_{i=1}^{N_{s}} \left(1+\frac{\left[S_{i}\right]^{\alpha_{i}}}{K_{S_{i}}^{\alpha_{i}}}\right)\times\prod_{j=1}^{N_{p}} \left(1+\frac{\left[P_{j}\right]^{\beta_{j}}}{K_{P_{j}}^{\beta_{j}}}\right)}$ (A8)

where *K_Si_* and *K_Pj_* are the binding constants (intrinsic model parameters) corresponding to substrates and products, respectively, and *V_maxf_* and *V_maxr_* are the forward and reverse reaction velocities (extrinsic model parameters).

### Thermodynamic constraints for enzymatic reactions:

To maintain the thermodynamic consistency of an enzymatic reaction, the forward and reverse reaction velocities are constrained by the Haldane relationship [2]. At equilibrium, the net reaction flux is zero (*J* = 0), which gives the following thermodynamic constraint for a N_S_-substrate N_P_-product enzymatic reaction:

$\frac{V_{maxf}\prod_{j=1}^{N_{p}} K_{P_{j}}^{\beta_{j}}}{V_{maxr}\prod_{i=1}^{N_{s}} K_{S_{i}}^{\alpha_{i}}}=\frac{\prod_{j=1}^{N_{p}} [P_{j}]_{eq}^{\beta_{j}}}{\prod_{i=1}^{N_{s}} [S_{i}]_{eq}^{\alpha_{i}}}=K'_{eq}$ (A9)

where $K'_{eq}$ is the apparent equilibrium constant for the reaction, which is the value of the reaction equilibrium-state quotient (i.e. ratio of product of product concentrations over product of substrate concentrations) at specified thermodynamic conditions (i.e. temperature, ionic strength, and pH). The apparent equilibrium constant at pH = 7 ($K'^{0}_{eq}=K'_{eq}(\text{pH}=7))$ is calculated as:

$K'^{0}_{eq}=e^{-\text{Δ}_{r}G'^{0}/RT}$ (A10)

where $\text{Δ}_{r}G'^{0}$, *R* and *T* are the standard Gibbs free energy of the reaction at pH = 7, gas constant, and temperature, respectively. The standard Gibbs free energy of reactions in the mitochondrial bioenergetics model was obtained from references [2-4], and are listed in Part C.

With the Haldane constraint (A9), *V_maxr_* can be calculated from *V_maxf_* and $K'^{0}_{eq}$ to reduce the number of unknown model parameters. Thus, the reaction flux expression for multi-substrate and multi-product enzymatic reaction (Eq A8) becomes:

$J=\frac{\frac{V_{maxf}}{\prod_{i=1}^{N_{s}} K_{S_{i}}^{\alpha_{i}}}\left(\prod_{i=1}^{N_{s}} \left[S_{i}\right]^{\alpha_{i}}-\frac{\prod_{j=1}^{N_{p}} \left[P_{j}\right]^{\beta_{j}}}{K'_{eq}}\right)}{\prod_{i=1}^{N_{s}} \left(1+\frac{\left[S_{i}\right]^{\alpha_{i}}}{K_{S_{i}}^{\alpha_{i}}}\right)\times\prod_{j=1}^{N_{p}} \left(1+\frac{\left[P_{j}\right]^{\beta_{j}}}{K_{S_{j}}^{\beta_{j}}}\right)}$ (A11)

Under the assumption that the substrate and product representing a co-factor pair bind with a given enzyme at the same binding site, one can show that the resulting reaction flux equation does not include the corresponding substrate and product terms. Since in the proposed integrated bioenergetics model all the co-factor pairs (e.g. NADH/NAD^+^, ATP/ADP/Pi, GTP/GDP/Pi and CoA/ACoA/SCoA) are assumed to bind to a given enzyme at the same binding site, the reaction flux equation for the enzyme is modified accordingly. Detailed reaction fluxes of all the enzymatic reactions in the mitochondrial bioenergetics model are given in Part C.

### Metabolite transporters:

Most of the metabolite transporters in the proposed integrated bioenergetics model involve the transport of two different metabolites or a metabolite and an ion. In general, there are two different types of such transporters: a co-transporter (symporter) that transports two different substances from one side to the other side of the inner mitochondrial membrane (IMM), and an antiporter (exchanger) that transports two different substances across the IMM in the opposite directions [1]. There are also uniporters, which transport one substance across the IMM, such as a Ca^2+^ uniporter, which is not considered in the present integrated bioenergetics model.

For generality, a random-ordered rapid-equilibrium binding mechanism is assumed for all the transporters, i.e., the transporter can bind to the two substrates in an arbitrary order, similar to that described for a reversible two-substrate two-product enzymatic reaction. Thus, the transport flux equations for various transporters can be derived similarly.

The general scheme for a co-transporter is shown below in Fig A3:

Fig A3. General scheme for a co-transporter

Here $A_{i}$ and $B_{i}$ are two distinct substrates at side i of the membrane (*i* =1, 2). The free transporter (*E*) binds to the two substrates in an arbitrary order before undergoing conformational changes. All association and dissociation processes are assumed to be reversible and rapid-equilibrium reactions. The rate limiting steps are the conformational changes in the substrate-bound transporters resulting in the transport of the substrates across the membrane.

Assuming rapid equilibrium, the general flux equation for a co-transporter is given by

$J_{co}=\frac{T_{\max f}\frac{\left[A_{1}\right]\left[B_{1}\right]}{K_{A1}K_{B1}}-T_{\max r}\frac{\left[A_{2}\right]\left[B_{2}\right]}{K_{A2}K_{B2}}}{1+\frac{\left[A_{1}\right]}{K_{A1}}+\frac{\left[A_{2}\right]}{K_{A2}}+\frac{\left[B_{1}\right]}{K_{B1}}+\frac{\left[B_{2}\right]}{K_{B2}}+\frac{\left[A_{1}\right]\left[B_{1}\right]}{K_{A1}K_{B1}}+\frac{\left[A_{2}\right]\left[B_{2}\right]}{K_{A2}K_{B2}}}$ (A12)

where *T_maxf_* and *T_maxr_* are the maximum forward and reverse transport rates, respectively.

The general scheme for an anti-porter is shown below in Fig A4:

Fig A4. General scheme for an anti-porter

The anti-porters bind to the substrates from both sides of the membrane. The general flux equation for an anti-porter is similarly given by:

$J_{anti}=\frac{T_{\max f}\frac{\left[A_{1}\right]\left[B_{2}\right]}{K_{A1}K_{B2}}-T_{\max r}\frac{\left[A_{2}\right]\left[B_{1}\right]}{K_{A2}K_{B1}}}{1+\frac{\left[A_{1}\right]}{K_{A1}}+\frac{\left[A_{2}\right]}{K_{A2}}+\frac{\left[B_{1}\right]}{K_{B1}}+\frac{\left[B_{2}\right]}{K_{B2}}+\frac{\left[A_{1}\right]\left[B_{2}\right]}{K_{A1}K_{B2}}+\frac{\left[A_{2}\right]\left[B_{1}\right]}{K_{A2}K_{B1}}}$ (A13)

For all the metabolite transporters, the internal and external binding constants are assumed to be equal (*K_A1_* = *K_A2_* and *K_B1_* = *K_B2_*). Therefore:

$J_{co}=\frac{\frac{T_{\max f}}{K_{A}K_{B}}\left(\left[A_{1}\right]\left[B_{1}\right]-\frac{\left[A_{2}\right]\left[B_{2}\right]}{K_{eq}}\right)}{1+\frac{\left[A_{1}\right]}{K_{A}}+\frac{\left[A_{2}\right]}{K_{A}}+\frac{\left[B_{1}\right]}{K_{B}}+\frac{\left[B_{2}\right]}{K_{B}}+\frac{\left[A_{1}\right]\left[B_{1}\right]}{K_{A}K_{B}}+\frac{\left[A_{2}\right]\left[B_{2}\right]}{K_{A}K_{B}}}$ (A14)

$J_{anti}=\frac{\frac{T_{\max f}}{K_{A}K_{B}}\left(\left[A_{1}\right]\left[B_{2}\right]-\frac{\left[A_{2}\right]\left[B_{1}\right]}{K_{eq}}\right)}{1+\frac{\left[A_{1}\right]}{K_{A}}+\frac{\left[A_{2}\right]}{K_{A}}+\frac{\left[B_{1}\right]}{K_{B}}+\frac{\left[B_{2}\right]}{K_{B}}+\frac{\left[A_{1}\right]\left[B_{2}\right]}{K_{A}K_{B}}+\frac{\left[A_{2}\right]\left[B_{1}\right]}{K_{A}K_{B}}}$ (A15)

For non-electrogenic transporters, *T_maxf_ =T_maxr_* and *K_eq_* = 1. For electrogenic transporters, such as the glutamate-aspartate exchanger (GAE) and the adenine nucleotide translocase (ANT), equilibrium constants are dependent on membrane potential, determined based on the Nernst equation. Detailed transport flux expressions for such transporters are described in Part C.

## Part C: Flux expressions for metabolic reactions and transport processes

In the proposed mitochondrial bioenergetics model, metabolic reaction and transport flux equations are derived based on thermodynamically-constrained enzymatic reaction and transport kinetic mechanisms [1, 5]. As described below, the intrinsic model parameters, such as binding constants (*K’*s), were either determined using previously published isolated enzyme and transporter kinetic data or set to well-established published values. Most of the enzyme kinetic data were measured using isolated mitochondrial enzymes purified from the heart or liver tissue, as no such kinetic data are available for lung tissue. The assumption is that differences in the intrinsic properties of the mitochondrial enzymes, such as binding constants (*K’*s), between the heart, liver and lung are relatively small [5], under fixed experimental conditions (temperature, pH, and ionic strength). With the intrinsic model parameters *K’*s known, the extrinsic model parameters, such as the maximum reaction and transport velocities (*V_max_*s and *T_max_*s), of the integrated bioenergetics model for isolated lung mitochondria were then estimated using previously published and newly measured experimental data from isolated rat lung mitochondria as described in the Results section of the main paper. The extrinsic model parameters are assumed to be dependent on the activities of the enzymes and transporters in a particular tissue, which is dependent on several cellular factors, such as enzyme and transporter expressions, concentrations, post-translational modifications, and physiological state of the tissue. Particularly the mitochondrial enzyme and transporter activities in non-excitable lung tissue is expected to be significantly different from that in excitable cardiac and skeletal muscle tissues.

In what follows, we provide the flux expressions for different enzymes and transporters used in the proposed integrated bioenergetics model. These flux expressions are determined based on the generalized flux expressions derived in the previous section (Part B). These flux expressions are then fitted to available experimental data on the kinetics of specific enzymes and transporters to estimate the associated parameter values (i.e. *K*’s, *V*_max_’s, *T*_max_’s). This is done using the “*fmincon*” algorithm in the MatLab optimization toolbox. Kinetic parameters for some of the enzymes and transporters are fixed at the well-established published values for which suitable kinetic data could not be identified. Only the values (estimated or fixed) of the intrinsic model parameters, such as binding constants *K*’s, are used in the integrated bioenergetics model. All extrinsic model parameters, such maximal reaction and transport velocities (*V*_max_’s and *T*_max_’s), were re-estimated based on fittings of the integrated bioenergetics model to available and new experimental data from isolated rat lung mitochondrial experiments (Fig 1-3 in the main paper).

### Reaction 1: Pyruvate dehydrogenase (PDH)

### PYR_m_ + CoA_m_ + NAD_m_ ⇌ ACoA_m_ + CO_2_ + NADH_m_ + H_m_^+^

For this reaction, the reactant concentrations are denoted as A = $[PYR_{m}]$, B = $[CoA_{m}]$, C = $[NAD_{m}]$, D = $[ACoA_{m}]$, E = $[CO_{2,m}]$, F = $[NADH_{m}]$. CO_2_ concentration is set at 1.32×10^-5^ M based on Henry’s law and is assumed to be constant. The participating co-factor pairs in the above reaction are NADH/NAD and CoA/ACoA. Thus, the overall reaction flux equation, $J_{PDH}$, is given by

$J_{PDH}=\frac{\frac{V_{maxf}}{K_{A}K_{B}K_{C}}\left(ABC-\frac{DEF}{K'_{eq}}\right)}{\left(1+\frac{A}{K_{A}}\right)\left(1+\frac{B}{K_{B}}+\frac{D}{K_{D}}\right)\left(1+\frac{C}{K_{C}}+\frac{F}{K_{F}}\right)\left(1+\frac{E}{K_{E}}\right)}$ (A16)

The above PDH-catalyzed reaction involves the generation of one proton. Thus, the pH-dependent apparent equilibrium constant for this reaction is defined as:

$K'_{eq}=e^{-\text{Δ}_{r}G_{PDH}^{'0}/RT}\times10^{pH_{m}-7}$ (A17)

where $pH_{m}$ is pH in the mitochondrial matrix, and$\Delta_{r}G'_{PDH}^{0}$ is the transformed Gibbs free energy of the reaction catalyzed by PDH at pH = 7. Since the CO_2_ concentration is assumed constant, the factor $\left(1+E/K_{E}\right)$ in the denominator reduces to a constant value.

Fig A5 shows the PDH reaction flux measured in isolated enzyme over a range of PYR, CoA, or NAD concentrations [6]. The model parameters (Table A4) were estimated by simultaneously fitting the solution of Eq A16 to all the data in Fig A5 using the MATLAB “*fmincon*” optimizer. The solid lines in Fig A5 are the fits of Eq A16 to the data.

Even though the binding constant of PYR for PDH is estimated to be ~25 μM in isolated enzyme experiments (as shown in Fig A5A and Table A4), the experimental data (Fig 2 in the main paper) at the whole mitochondria level show that the apparent binding constant of PYR for PDH is around ~3-5 mM. This apparent inconsistency is caused by the enzyme pyruvate dehydrogenase kinase (PDK) [5]. PDH is known to be inhibited by the PDK, and PDK is inhibited by PYR. Thus, the apparent binding constant of PYR for PDH is affected by PDK. Therefore, for simplicity, only the apparent binding constant of PYR for PDH is used for the simulations of the integrated bioenergetics model in the main paper.

Table A4. PDH model kinetic parameters

| **Parameters** | **Definition** | **Value** | **Source** |
| --- | --- | --- | --- |
| *V*_maxf_ | Maximum forward reaction rate | 96.8 nmol/min/mg enzyme | Estimated using data in Fig A5 |
| $K_{A}$ | PYR binding constant in isolated PDH* | $2.52\times10^{-5} M$ | Estimated using data in Fig A5 |
| $K_{B}$ | CoA binding constant | $1.49\times10^{-5} M$ | Estimated using data in Fig A5 |
| $K_{C}$ | NAD binding constant | $3.50\times10^{-5} M$ | Estimated using data in Fig A5 |
| $K_{D}$ | ACoA binding constant | $1.49\times10^{-5} M$ | Assumed to be the same as *K_B_* |
| $K_{F}$ | NADH binding constant | $3.50\times10^{-5} M$ | Assumed to be the same as *K_C_* |
| $\text{Δ}_{r}G_{PDH}^{'0}$ | Gibbs free energy of the reaction (pH=7) | -38.64 kJ/mol | [4] |

*The binding constant of PYR for PDH is affected by the enzyme pyruvate dehydrogenase kinase (PDK). Thus only the apparent binding constant is used for the model simulations in the main paper.

**Fig A5.** Symbols: Reaction fluxes of pyruvate dehydrogenase (PDH) measured in isolated PDH in reference [6]. In these experiments, around ~16-20$\mu g$ PDH enzyme was used to catalyze the reaction. Reaction fluxes are plotted with three substrates varying at the concentrations as shown in the figures. **(A)** PYR concentration was varied from 0 to 0.6 mM, with CoA concentration fixed at 0.2 mM (red), 0.1 mM (blue), 0.07 mM (green), or 0.05 mM (cyan), respectively; NAD concentration was fixed at 0.05 mM. **(B)** CoA concentration was varied from 0 to 0.3 mM, with NAD concentration fixed to 0.5 mM (red), 0.25mM (blue), 0.1 mM (green), or 0.05 mM (cyan), respectively; PYR concentration was fixed at 0.25mM. **(C)** NAD concentration was varied from 0 to 0.6 mM, with PYR concentration fixed at 0.5 mM (red), 0.25 mM (blue), 0.1 mM (green), or 0.05 mM (cyan), respectively; CoA concentration was fixed at 0.1 mM. The solid lines are the fits of Eq A16 to the data.

### Reaction 2: Citrate synthase (CITS)

### ACoA_m_+OXA_m_ ⇌ CoA_m_+ CIT_m_ +2H_m_^+^

For this reaction, the reactant concentrations are denoted as A = $[ACoA_{m}]$, B = $[OXA_{m}]$, C = $[CoA_{m}]$, D = $[CIT_{m}]$. Since CoA and ACoA are co-factor pairs, the overall reaction flux equation,$J_{CITS}$, is given by

$J_{CITS}=\frac{\frac{V_{maxf}}{K_{A}K_{B}}\left(AB-\frac{CD}{K'_{eq}}\right)}{\left(1+\frac{A}{K_{A}}+\frac{C}{K_{C}}\right)\left(1+\frac{B}{K_{B}}\right)\left(1+\frac{D}{K_{D}}\right)}$ (A18)

The reaction catalyzed by CITS generates two protons. Thus, the pH-dependent apparent equilibrium constant for this reaction is defined as:

$K'_{eq}=e^{-\text{Δ}_{r}G_{CITS}^{'0}/RT}\times10^{2pH_{m}-14}$ (A19)

Fig A6 shows the CITS reaction flux measured in isolated enzyme over a range of ACoA, OXA, CIT, or CoA concentrations [7]. The model parameters in Table A5 were estimated by simultaneously fitting the solution of Eq A18 to all the data in Fig A6 using the MATLAB “*fmincon*” optimizer. The solid lines in Fig A6 are the fits of Eq A18 to the data.

**Fig A6.** **(A)** Forward flux as a function of substrates ACoA and OXA. The concentrations of OXA was 2.5 $\mu$M (green), 5.0 $\mu$M (blue), or 10 $\mu M$ (red). **(B)** Forward reaction flux as a function of substrates OXA and ACoA. ACoA concentration was 2.5$\mu$M (green), 5.0$\mu$M (blue), or 10$\mu M$ (red). **(C)** Reverse reaction flux as a function of CoA and CIT. CoA concentration was 20 $\mu$M (green), 33 $\mu$M (cyan), 50 $\mu$M (blue), or 100 $\mu$M (red). **(D)** Reverse reaction flux as a function of concentrations of CoA and CIT. CIT concentration was 1.0 mM (green), 2.0 mM (cyan), 4.0 mM (blue), or 10 mM (red). This reaction was fit using data from reference [7] on forward reaction fluxes (Figs A6A and A6B) and reverse reaction fluxes (Figs A6C and A6D). The solid lines are the fit of Eq A18 to the data. All data were obtained at 28°C, pH = 8.1.

Table A5. CITS model kinetic parameters

| **Parameters** | **Definition** | **Value** | **Source** |
| --- | --- | --- | --- |
| V_maxf_ | Maximum forward reaction rate | 243 nmol/min/mg enzyme | Estimated using data in Fig A6 |
| $K_{A}$ | ACoA binding constant | $3.90\times10^{-6} M$ | Estimated using data in Fig A6 |
| $K_{B}$ | OXA binding constant | $4.53\times10^{-6} M$ | Estimated using data in Fig A6 |
| $K_{C}$ | CoA binding constant | $57.9\times10^{-6} M$ | Estimated using data in Fig A6 |
| $K_{D}$ | CIT binding constant | $4.30\times10^{-3} M$ | Estimated using data in Fig A6 |
| $\text{Δ}_{r}G_{CITS}^{'0}$ | Gibbs free energy of the reaction (pH=7) | -36.61kJ/mol | [4] |

### Reaction 3: Aconitase and iso-citrate dehydrogenase (CITDH)

The reactions catalyzed by aconitase and isocitrate (ISOCIT) dehydrogenase are:

*Aconitase:* CIT ⇌ ISOCIT + H_2_O

*Isocitrate dehydrogenase:* ISOCIT +NAD + H_2_O ⇌ AKG + NADH + CO_2_

Under the assumption that the reaction catalyzed by aconitase is rapidly equilibrating, the reactions catalyzed by aconitase and isocitrate can be lumped into the following reaction

CIT_m_ +NAD_m_ ⇌ AKG_m_ + NADH_m_ +CO_2_

For this reaction, the reactant concentrations are denoted as A = $[CIT_{m}]$, B = $[NAD_{m}]$, C = $[AKG_{m}]$, D = $[NADH_{m}]$, and E = $[CO_{2, m}]$, which is assumed to be constant.

$J_{CITDH}=\frac{\frac{V_{maxf}}{K_{A}K_{B}}\left(AB-\frac{CDE}{K'_{eq}}\right)}{\left(1+\frac{A}{K_{A}}\right)\left(1+\frac{C}{K_{C}}\right)\left(1+\frac{B}{K_{B}}+\frac{D}{K_{D}}\right)\left(1+\frac{E}{K_{E}}\right)}$ (A20)

The production of CO_2_ results in the generation of bicarbonate ions and one proton. Thus, the equilibrium constant for this reaction is defined as:

$K'_{eq}=e^{-\text{Δ}_{r}G_{CITDH}^{'0}/RT}10^{pH_{m}-7}$ (A21)

Fig A7 shows the CITDH reaction flux measured in isolated enzyme over a range of NAD or NADH concentrations. The model parameters in Table A6 were estimated by simultaneously fitting the solution of Eq A20 to the data in Fig A7 using the MATLAB “*fmincon*” optimization program package.

**Fig A7.**  **Symbols are reaction fluxes of isocitrate dehydrogenase measured in isolated enzyme experiments from reference [8].** Reaction fluxes are plotted with NAD and NADH concentrations varying at the concentrations shown in the figure. **(A)** Enzyme activity as a function of NAD in the absence (blue, triangle) or presence of 0.039 mM NADH (red, asterisk). **(B)** Inhibition effect of NADH on enzyme activity. Percent inhibition was calculated as 100(v_0_-v)/v_0_, where v_0_ and v are the enzyme flux rates in the absence or presence of NADH, respectively. The solid lines are model fits of Eq A20 to the data.

**Table A6. CITDH model kinetic parameters**

| **Parameters** | **Definition** | **Value** | **Source** |
| --- | --- | --- | --- |
| V_maxf_ | Maximum forward reaction rate | $3.6\times10^{6}$nmol  /min/mg enzyme | Estimated using data in Fig A7 |
| K_A_ | CIT binding constant | $1.30\times10^{-3} M$ | Estimated using data in Fig A7 |
| K_B_ | NAD binding constant | $500\times10^{-6} M$ | Estimated using data in Fig A7 |
| K_C_ | AKG binding constant | $3.50\times10^{-6} M$ | Estimated using data in Fig A7 |
| K_D_ | NADH binding constant | $4.70\times10^{-6} M$ | Estimated using data in Fig A7 |
| $\text{Δ}_{r}G_{CITDH}^{'0}$ | Gibbs free energy of the reaction (pH=7) | 2.81 KJ/mol | [4] |

### Reaction 4: AKG dehydrogenase (AKGDH)

### AKG_m_ + CoA_m_ + NAD_m_ ⇌ SCoA_m_+NADH_m_+CO_2_

For this reaction, the reactant concentrations are denoted as A =$[AKG_{m}]$, B = $[CoA_{m}]$, C = $[NAD_{m}]$, D = $[SCoA_{m}]$, E = $[NADH_{m}]$, and F = $[CO_{2}]$. The participating co-factor pairs in the above reaction are CoA/ACoA, and NADH/NAD. Thus, the overall reaction flux equation $J_{AKGDH}$ is given below.

$J_{AKGDH}=\frac{\frac{V_{maxf}}{K_{A}K_{B}K_{C}}\left(ABC-\frac{DEF}{K'_{eq}}\right)}{\left(1+\frac{C}{K_{C}}+\frac{E}{K_{E}}\right)\left(1+\frac{B}{K_{B}}+\frac{D}{K_{D}}\right)\left(1+\frac{A}{K_{A}}\right)}$ (A22)

Since the CO_2_ produced results in the generation of a bicarbonate ion and one proton, the equilibrium constant for this reaction is defined as

$K'_{eq}=e^{-\text{Δ}_{r}G_{AKGDH}^{'0}/RT}10^{pH_{m}-7}$ (A23)

Fig A8 shows the AKGDH flux measured in isolated enzyme over a range of AKG, COA or NAD concentrations. The model parameters in Table A7 were estimated by simultaneously fitting the solution of Eq A22 to the data in Fig A8 using the MATLAB “*fmincon*” optimization program package. The solid lines in Fig A8 are the fits of Eq A22 to the data.

Table A7. AKGDH model kinetic parameters

| **Parameters** | **Definition** | **Value** | **Source** |
| --- | --- | --- | --- |
| *V_maxf_* | Maximum forward reaction rate | $1.07\times10^{3}$nmol/min/mg  enzyme | Estimated using data in Fig A8 |
| *K_A_* | AKG binding constant | $85.9\times10^{-6} M$ | Estimated using data in Fig A8 |
| *K_B_* | CoA binding constant | $1.63\times10^{-6} M$ | Estimated using data in Fig A8 |
| *K_C_* | NAD binding constant | $46.6\times10^{-6} M$ | Estimated using data in Fig A8 |
| *K_D_* | SCoA binding constant | $1.63\times10^{-6} M$ | Assumed the same as *K_B_* |
| *K_E_* | NADH binding constant | $7.14\times10^{-6} M$ | Estimated using data in Fig A8 |
| $\text{Δ}_{r}G_{AKGDH}^{'0}$ | Gibbs free energy of the reaction (pH=7) | -37.08 KJ/mol | [4] |

**Fig A**8**. AKGDH activity as a function of substrate and product concentrations.** **(A)** Forward reaction fluxes as a function of AKG concentration as shown at x axis. And concentrations of NAD and CoA were set to 0.033 mM and 0.005 mM (cyan), 0.066mM and 0.01 mM (blue), 0.133 mM and 0.02 mM (green), 0.333 mM and 0.05 mM (red). **(B)** Forward reaction fluxes as a function of CoA concentration shown on the x axis. The concentrations of AKG and NAD were set to 0.025 mM and 0.02 mM (cyan), 0.05 mM and 0.04 mM (blue), 0.1 mM and 0.08 mM (green), 0.5 mM and 0.4 mM (red). **(C)** Forward reaction fluxes as a function of NAD concentration shown on the x axis. The concentrations of AKG and CoA were set to 0.05 mM and 0.005 mM (cyan), 0.1 mM and 0.01mM (blue), 0.2 mM and 0.02 mM (green), 0.5 mM and 0.05 mM (red). **(D)** Net reaction fluxes as a function of NAD concentration shown on the x axis. AKG and CoA concentrations were fixed to 0.5 mM and 0.05 mM, respectively. The NADH concentration was set to 0 mM (red), 0.01 mM (green), 0.02 mM (blue), 0.05 mM (cyan). The symbols are experimental data [9] and solid lines are model fits to the data.

### Reaction 5: SCoA synthetase (SCAS)

SCoA_m_ + GDP_m_ + Pi_m_ ⇌ SUC_m_ + GTP_m_ + CoA_m_ + H_m_^+^

For this reaction, the reactant concentrations are denoted as A = $[SCoA_{m}]$, B = $[GDP_{m}]$, C = $[Pi_{m}]$, D = $[SUC_{m}]$, E =$[GTP_{m}]$, and F = $[CoA_{m}]$. The participating co-factor pairs in this reaction are SCoA/CoA, ADP/ATP, ATP/Pi, and SCoA/SUC. Therefore, the overall reaction flux equation $\Phi_{SCAS}$ is given by:

$J_{SCAS}=\frac{\frac{V_{maxf}}{K_{A}K_{B}K_{C}}\left(ABC-\frac{DEF}{K'_{eq}}\right)}{\left(1+\frac{A}{K_{A}}+\frac{D}{K_{D}}+\frac{F}{K_{F}}+\frac{DF}{K_{D}K_{F}}\right)\left(1+\frac{B}{K_{B}}+\frac{C}{K_{C}}+\frac{E}{K_{E}}+\frac{BC}{K_{B}K_{C}}\right)}$ (A24)

This reaction involves the generation of one proton, thus, the equilibrium constant for this reaction is defined as

$K'_{eq}=e^{-\text{Δ}_{r}G_{SCAS}^{'0}/RT}10^{pH_{m}-7}$ (A25)

Fig A9 shows the SCAS flux measured in isolated enzyme over a range of GTP, GDP or SCA concentrations. The model parameters in Table A8 were estimated by simultaneously fitting the solution of Eq A24 to the data in Fig A9 using the MATLAB “*fmincon*” optimization program package. The solid lines in Fig A9 are the fits of Eq A24 to the data.

Table A8. SCAS model kinetic parameters

| **Parameters** | **Definition** | **Value** | **Source** |
| --- | --- | --- | --- |
| *V_maxf_* | Maximum forward reaction rate | 50.6 nmol/min/mg enzyme | Estimated using data in Fig A9 |
| *K_A_* | SCoA binding constant | $12.5\times10^{-6} M$ | Estimated using data in Fig A9 |
| *K_B_* | GDP binding constant | $5.70\times10^{-6} M$ | Estimated using data in Fig A9 |
| *K_C_* | Pi binding constant | $2.10\times10^{-3} M$ | Estimated using data in Fig A9 |
| *K_D_* | SUC binding constant | $451\times10^{-6} M$ | Estimated using data in Fig A9 |
| *K_E_* | GTP binding constant | $23.1\times10^{-6} M$ | Estimated using data in Fig A9 |
| *K_F_* | CoA binding constant | $17.7\times10^{-6} M$ | Estimated using data in Fig A9 |
| $\text{Δ}_{r}G_{SCAS}^{'0}$ | Gibbs free energy of the reaction (pH=7) | 1.26 KJ/mol | [4] |

**Fig A9.** **SCAS activity as a function of substrate and product concentrations [10].** **(A)** Reverse reaction fluxes as a function of GTP concentration shown on the x axis. SUC concentrations were fixed at 0.4 mM (red), 1 mM (green), 10 mM (blue), 50 mM (cyan), while CoA was fixed to 0.1 mM. **(B)** Forward reaction fluxes as a function of GDP concentration as shown at x axis. The concentrations of GTP was set to 0 mM (red), 0.05 mM (green) and 0.1 mM (blue), while inorganic phosphate (Pi) concentration fixed at 50 mM and SCoA concentration fixed at 0.1mM. **(C)** Forward reaction fluxes as a function of SCoA concentration shown on the x axis. The concentrations of CoA were set to 0 mM (red), 0.02 mM (green), 0.04 mM (blue). **(D)** Forward reaction fluxes as a function of SCoA concentration shown on the x axis. SUC concentration were fixed to 0 mM (blue), 0.5 mM (green), 2 mM (red). While phosphate concentration and GDP concentrations are fixed at 1 mM and 0.05 mM, respectively.

### Reaction 6: Nucleoside diphosphokinase (NDK)

GTP_m_+ADP_m_ ⇌ GDP_m_+ATP_m_

For this reaction, the reactant concentrations are denoted as A =$[GTP_{m}]$, B = $[ADP_{m}]$, C = $[GDP_{m}]$, and D = $[ATP_{m}]$, and the overall reaction flux equation is given by:

$J_{NDK}=\frac{\frac{V_{maxf}}{K_{A}K_{B}}\left(AB-\frac{CD}{K'_{eq}}\right)}{\left(1+\frac{A}{K_{A}}+\frac{C}{K_{C}}\right)\left(1+\frac{B}{K_{B}}+\frac{D}{K_{D}}\right)}$ (A26)

Table A9. NDK model kinetic parameters

| **Parameters** | **Definition** | **Value** | **Source** |
| --- | --- | --- | --- |
| *K_A_* | GTP binding constant | $111\times10^{-6}$ M | [2] |
| *K_B_* | ADP binding constant | $100\times10^{-6}$ M | [2] |
| *K_C_* | GDP binding constant | $260\times10^{-6}$ M | [2] |
| *K_D_* | ATP binding constant | $278\times10^{-6}$ M | [2] |
| $\text{Δ}_{r}G_{NDK}^{'0}$ | Gibbs free energy of the reaction (pH=7) | 0 KJ/mol | [4] |

Parameters in this reaction do not affect the simulations in the main paper since the reaction is rapidly equilibrating.

### Reaction 7: Succinate dehydrogenase (SDH)

### SUC_m_ + FAD_m_ ⇌ FUM_m_ + FADH_2,m_,

For this reaction, the reactant concentrations are denoted as A = $[SUC_{m}]$, B = [$FAD_{m}$], C = $[FUM_{m}]$, and D =$[FADH_{2,m}]$, and the participating co-factor pairs are FAD and FADH_2_. The overall reaction flux equation is given by:

$J_{SDH}=\frac{\frac{V_{maxf}}{K_{A}K_{B}}\left(AB-\frac{CD}{K'_{eq}}\right)}{\left(1+\frac{A}{K_{A}}\right)\left(1+\frac{C}{K_{C}}\right)\left(1+\frac{B}{K_{B}}+\frac{D}{K_{D}}\right)}$ (A27)

Fig A10 shows reaction fluxes for different concentrations of SUC and the exogenous electron acceptor phenazine methosulphate. The model parameters in Table A10 were estimated by simultaneously fitting the solution of Eq A27 to the data in Fig A10 using the MATLAB “*fmincon*” optimization program package. The solid lines in Fig A10 are the fits of Eq A27 to the data.

**Fig A10. SDH activity as a function of succinate and electron acceptor (phenazine methosulphate) concentrations [11].** Succinate concentration is indicated on the x axis, and electron acceptor concentrations are 0.067 (red), 0.10 (blue), 0.167 (yellow), 0.30 (cyan), and 2 mM (black), respectively.

Table A10. SDH model kinetic parameters

| **Parameters** | **Definition** | **Value** | **Source** |
| --- | --- | --- | --- |
| *V_maxf_* | Maximum forward reaction rate | $1.20\times10^{-6}\Delta_{600} /\min$ | Estimated using data in Fig A10 |
| *K_A_* | SUC binding constant | $1.80\times10^{-3} M$ | Estimated using data in Fig A10 |
| *K_B_* | FAD mimic binding constant | $140\times10^{-6} M$ | Estimated using data in Fig A10 |
| *K_C_* | FUM binding constant | $1.80\times10^{-3} M$ | Assumed the same as *K_A_* |
| *K_D_* | FADH_2_ binding constant | $2.45\times10^{-6} M$ | Estimated using data in Fig A10 |
| $\text{Δ}_{r}G_{SDH}^{'0}$ | Gibbs free energy of the reaction (pH=7) | -3.62 KJ/mol | [4] |

### Reaction 8: Fumarate hydratase (FH)

|  | FUM_m_ + H_2_O ⇌ MAL_m_ |  |
| --- | --- | --- |

For this rapidly equilibrating reaction, the reactant concentrations are denoted as A = $[FUM_{m}]$, and B = $[MAL_{m}]$. The reactant H_2_O is ignored in this reaction since its concentration is assumed to be constant.

$J_{FUM}=\frac{\frac{V_{maxf}}{K_{A}K_{B}}\left(A-\frac{B}{K'_{eq}}\right)}{\left(1+\frac{A}{K_{A}}\right)\left(1+\frac{B}{K_{B}}\right)}$ A28)

Table A11. FH model kinetic parameters

| **Parameters** | **Definition** | **Value** | **Source** |
| --- | --- | --- | --- |
| *K_A_* | FUM binding constant | $1.8\times10^{-3} M$ | fixed |
| *K_B_* | MAL binding constant | $1.8\times10^{-3} M$ | fixed |
| $\text{Δ}_{r}G_{FH}^{'0}$ | Gibbs free energy of the reaction (pH=7) | -3.6 KJ/mol | [4] |

### Reaction 9: Malate dehydrogenase (MDH)

### MAL_m_ + NAD_m_ ⇌ OXA_m_ + NADH_m_ + H_m_^+^

For this reaction, the reactant concentrations are denoted as A = $[NAD_{m}]$, B = $[MAL_{m}]$, C = $[OXA_{m}]$, D = $[NADH_{m}]$, the participating co-factor pairs are NADH/NAD, and MAL is known to be competitive inhibitor of OXA [12]. Thus, the overall reaction flux equation is given by:

$J_{MDH}=\frac{\frac{V_{maxf}}{K_{A}K_{B}}\left(AB-\frac{CD}{K'_{eq}}\right)}{\left(1+\frac{B}{K_{B}}+\frac{C}{K_{C}}\right)\left(1+\frac{A}{K_{A}}+\frac{D}{K_{D}}\right)}$ (A29)

The above reaction involves the generation of one proton, thus, the equilibrium constant for this reaction is defined as

$K'_{eq}=e^{-\text{Δ}_{r}G_{MDH}^{'0}/RT}10^{pH_{m}-7}$ (A30)

Fig A11 shows reaction fluxes over different concentrations of NAD, OXA, and NADH. The model parameters in Table A12 were estimated by simultaneously fitting the solution of Eq A29 to the data in Fig A11 using the MATLAB “*fmincon*” optimization program package. The solid lines in Fig A11 are the fits of Eq A29 to the data.

MAL

MAL

NADH

**Fig A11. MDH activity [12] as a function of substrate and product concentrations.** **(A)** Forward reaction flux as a function of NAD and MAL. NAD concentrations are indicated on the x axis. MAL concentrations are fixed to 0.33 mM (green), 0.5 mM (magenta), 0.67 mM (cyan), 1 mM (yellow), 2 mM (blue), 4 mM (red). Product concentrations are set to zero. **(B)** Reverse reaction flux as a function of OXA and NADH concentrations. NADH concentrations are fixed at 0.0067 mM (cyan), 0.015 mM (blue), 0.02 mM (green), 0.05 mM (red). **(C)** Reverse reaction flux as a function of NADH and MAL. NADH concentrations are indicated on the x axis and MAL concentrations were fixed to 0 mM (red), 1.5 mM (magenta), 3 mM (cyan), 4.5 mM (yellow), and 6 mM (blue).

Table A12. MDH model kinetic parameters

| **Parameters** | **Definition** | **Value** | **Source** |
| --- | --- | --- | --- |
| *V_maxf_* | Maximum forward reaction rate | 170 nmol/min/mg enzyme | Estimated using data in Fig A11 |
| *K_A_* | MAL binding constant | $155\times10^{-6} M$ | Estimated using data in Fig A11 |
| *K_B_* | NAD binding constant | $1.10\times10^{-3} M$ | Estimated using data in Fig A11 |
| *K_C_* | OXA binding constant | $3.38\times10^{-6} M$ | Estimated using data in Fig A11 |
| *K_D_* | NADH binding constant | $36.1\times10^{-6} M$ | Estimated using data in Fig A11 |
| $\text{Δ}_{r}G_{MDH}^{'0}$ | Gibbs free energy of the reaction (pH=7) | 28.83 KJ/mol | [4] |

### Reaction 10: Glutamate oxaloacetate transaminase (GOT)

ASP_m_ +AKG_m_ ⇌ GLU_m_ + OXA_m_

For this reaction, the reactant concentrations are denoted as A = $[ASP_{m}]$, B = $[AKG_{m}]$, C = $[GLU_{m}]$, D = $[OXA_{m}]$, and the overall reaction flux equation is given by:

$J_{GOT}=\frac{\frac{V_{maxf}}{K_{A}K_{B}}\left(AB-\frac{CD}{K'_{eq}}\right)}{\left(1+\frac{A}{K_{A}}\right)\left(1+\frac{B}{K_{B}}\right)\left(1+\frac{C}{K_{C}}\right)\left(1+\frac{D}{K_{D}}\right)}$ (A31)

The equilibrium constant for this reaction is:

$K'_{eq}=e^{-\text{Δ}_{r}G_{GOT}^{'0}/RT}$ (A32)

Table A13. GOT model kinetic parameters

| **Parameters** | **Definition** | **Value** | **Source** |
| --- | --- | --- | --- |
| *K_A_* | ASP binding constant | $3.9\times10^{-3} M$ | [2] |
| *K_B_* | ΑKG binding constant | $430\times10^{-6} M$ | [2] |
| *K_C_* | GLU binding constant | $8.9\times10^{-3} M$ | [2] |
| *K_D_* | OXA binding constant | $88\times10^{-6} M$ | [2] |
| $\text{Δ}_{r}G_{GOT}^{'0}$ | Gibbs free energy of the reaction (pH=7) | -1.31KJ/mol | [4] |

### Reaction 11: Complex I (CI)

### NADH_m_ + UQ_m_ + H_m_ ⇌ NAD_m_ + UQH_2m_ + 4ΔH

The above reaction catalyzed by complex I involves pumping four protons (ΔH) from the mitochondria matrix into the inter-membrane space. Thus, this reaction flux is dependent on proton motive force defined as $\Delta G_{H}=F\Delta\Psi+RTln\left([H_{i}^{+}]/[H_{m}^{+}]\right)$, where *F* is the Faraday’s constant and $\Delta\Psi$ is the mitochondrial membrane potential. To take this dependency into account, the kinetic parameters was modified to be dependent on membrane potential [13, 14].

For this reaction, the reactant concentrations are denoted as A = $[NADH_{m}]$, B = $[UQ_{m}]$, C = $[NAD_{m}]$, D = $[UQH_{2m}]$, and the participating co-factor pairs in this reaction are NAD/ NADH, and UQ/UQH_2_. Thus, the overall reaction flux equation is given by:

$\Phi_{CI}=\frac{\frac{V_{maxf}}{K_{A}K_{B}}\left\{\exp\left(\frac{-\beta_{CI}(4\Delta G_{H}+\text{Δ}_{r}G_{CI}^{'0})}{RT}\right)AB-\exp\left(\frac{-\left(\beta_{CI}-1\right)(4\Delta G_{H}+\text{Δ}_{r}G_{CI}^{'0})}{RT}\right)CD\right\}}{\left(1+\frac{A}{K_{A}}+\frac{C}{K_{C}}\right)\left(1+\frac{B}{K_{B}}+\frac{D}{K_{D}}\right)}$ (A33)

The model parameters in Table A14 were estimated by simultaneously fitting the solution of Eq A33 to the data in Fig A14 using the MATLAB “*fmincon*” optimization program package. The solid lines in Fig A14 are the fits of Eq A33 to the data.

**Fig A12. CI activity [15] as a function of substrate and product concentrations.** **(A)** NADH concentrations are varied as shown on the x axis. UQ concentrations was set to 25 µM (cyan), 50 µM (blue), 100 µM (green), 200 µM (red). Product concentrations are set to zero. **(B)** NADH concentrations are varied as shown on the x axis. UQ concentration was set to 100 µM and NAD concentration was fixed to 0 µM (red), 200 µM (green), 400 µM (blue), 600 µM (cyan). **(C)** NADH concentrations are varied as shown on the x axis. UQ concentrations was set to 100 µM and UQH_2_ concentrations were set to 0 µM (red), 100 µM (blue), 200 µM (green). **(D)** UQ concentrations are varied as shown on the x axis. NADH concentration was fixed to 10 µM and NAD concentration was set to 0 µM (red), 400 µM (blue), 600 µM (green).

Table A14. CI model kinetic parameters

| **Parameters** | **Definition** | **Value** | **Source** |
| --- | --- | --- | --- |
| V_maxf_ | Maximum forward reaction rate | 9.8 pmol/min/mg enzyme | Estimated using data in Fig A12 |
| K_A_ | NADH binding constant | $1.50\times10^{-6} M$ | Estimated using data in Fig A12 |
| K_B_ | UQ binding constant | $58.1\times10^{-6} M$ | Estimated using data in Fig A12 |
| K_C_ | NAD binding constant | $428\times10^{-6} M$ | Estimated using data in Fig A12 |
| K_D_ | UQH_2_ binding constant | $519\times10^{-6} M$ | Estimated using data in Fig A12 |
| β_CI_ | Complex I free energy barrier | 0.5 | Fixed |
| $\text{Δ}_{r}G_{CI}^{'0}$ | Gibbs free energy of the reaction (pH=7) | -69.37KJ/mol | [4] |

### Reaction 12: Complex II (CII)

### FADH_2,m_ + UQ_m_ ⇌ FAD_m_ + UQH_2,m_

For this reaction, the reactant concentrations are denoted as A = $[FADH_{2,m}]$, B = $[UQ_{m}]$, C = $[FAD_{m}]$, D = $[UQH_{2,m}]$, and the participating co-factor pairs are FAD/ FADH_2_, and UQ/UQH_2_. Thus, the overall reaction flux equation is given by:

$J_{CII}=\frac{\frac{V_{maxf}}{K_{A}K_{B}}\left(AB-\frac{CD}{K'_{eq}}\right)}{\left(1+\frac{A}{K_{A}}+\frac{C}{K_{C}}\right)\left(1+\frac{B}{K_{B}}+\frac{D}{K_{D}}\right)}$ (A34)

Table A15. CII model kinetic parameters

| **Parameters** | **Definition** | **Value** | **Source** |
| --- | --- | --- | --- |
| *K_A_* | FADH_2_ binding constant | $1.50\times10^{-6} M$ | Assume the same as NADH binding constant in CI |
| *K_B_* | UQ binding constant | $58.1\times10^{-6} M$ | Assume the same as UQ binding constant in CI |
| *K_C_* | FAD binding constant | $428\times10^{-6} M$ | Assume the same as NAD binding constant in CI |
| *K_D_* | UQH_2_ binding constant | $519\times10^{-6} M$ | Assume the same as UQH_2_ binding constant in CI |
| $\text{Δ}_{r}G_{CII}^{'0}$ | Gibbs free energy of the reaction (pH=7) | -1.31 KJ/mol | [4] |

### Reaction 13: Complex III (CIII)

### UQH_2m_ + 2CytC_o_ + 2H_m_ ⇌ UQ_m_ + 2CytC_r_ + 4ΔH

For this reaction, the reactant concentrations are denoted as A = $[UQH_{2,m}]$, B = $[CytCo]$, C = $[UQ_{m}]$, D = $[CytCr]$, and the participating co-factor pairs are UQ_m_/UQH_2,m_, and CytC_r_/CytC_o_. In addition, the above reaction catalyzed by complex III involves pumping four protons from mitochondria matrix into inter-membrane space. Thus, the overall reaction flux equation is given by:

$\Phi_{CIII}=\frac{\frac{V_{maxf}}{K_{A}K_{B}^{2}}\left(\begin{matrix} \exp\left\{\beta_{CIII}\left(\frac{-4\Delta G_{H}}{RT}+\frac{2F\Delta\Psi}{RT}-\frac{\text{Δ}_{r}G_{CIII}^{'0}}{RT}\right)\right\}AB^{2} \\ -\exp\left\{\left(\beta_{CIII}-1\right)\left(\frac{-4\Delta G_{H}}{RT}+\frac{2F\Delta\Psi}{RT}-\frac{\text{Δ}_{r}G_{CIII}^{'0}}{RT}\right)\right\}CD^{2} \end{matrix}\right)}{\left(1+\frac{A}{K_{A}}+\frac{C}{K_{C}}\right)\left(1+\frac{B^{2}}{K_{B}^{2}}+\frac{D^{2}}{K_{D}^{2}}\right)}$ (A35)

The model parameters in Table A16 were estimated by simultaneously fitting the solution of Eq A35 to the data in Fig A13 using the MATLAB “*fmincon*” optimization program package. The solid lines in Fig A13 are the fits of Eq A35 to the data.

Table A16. CIII model kinetic parameters

| **Parameters** | **Definition** | **Value** | **Source** |
| --- | --- | --- | --- |
| *V_maxf_* | Maximum forward reaction rate | 32 µmol/s/mg enzyme | Estimated using data in Fig A13 |
| *K_A_* | UQH_2_ binding constant | $4.66\times10^{-6} M$ | Estimated using data in Fig A13 |
| *K_B_* | CytCo binding constant | $3.76\times10^{-6} M$ | Estimated using data in Fig A13 |
| *K_C_* | UQ binding constant | $4.08\times10^{-6} M$ | Estimated using data in Fig A13 |
| *K_D_* | CytCr binding constant | $4.91\times10^{-6} M$ | Estimated using data in Fig A13 |
| *β_CIII_* | Complex III free energy barrier | 0.5 | Fixed |
| $\text{Δ}_{r}G_{CIII}^{'0}$ | Gibbs free energy of the reaction (pH=7) | -32.53KJ/mol | [4] |

**Fig A13. CIII activity [16] as a function of substrate and product concentrations as indicated in the figures.** **(A)** Oxidized form of cytochrome c concentrations were varied as shown on the x axis. UQH_2_ concentrations were set to 25 µM (cyan), 15 µM (blue), 10 µM (green), 7 µM (red). Product concentrations are set to zero. **(B)** Oxidized form of cytochrome c concentrations were varied as shown on the x axis. Reduced form of cytochrome c concentrations were set to 0 µM and 10 µM, respectively. UQ concentration was fixed to 20 µM. **(C)** UQH_2_ concentrations were varied as shown on the x axis. Reduced form of cytochrome c concentrations were set to 0 µM (cyan), 5 µM (blue), 10 µM (green), 15 µM (red). **(D)** UQH_2_ concentrations are varied as shown on the x axis. Oxidized form of cytochrome c concentrations were fixed to 7 µM (cyan), 12 µM (blue), 20 µM (green), 30 µM (red).

### Reaction 14: Complex IV (CIV)

### 2CytCr+0.5O_2_+2H_m_ ⇌ 2CytCo+H_2_O+2ΔH

For this reaction, the reactant concentrations are denoted as A = $[CytCr]$, B = $[O_{2}]$, C =$[CytCo]$, and the participating co-factor pair is CytCr/CytCo. In addition, two protons arepumped from the matrix side to the inter-membrane space. Another two protons are consumed in the matrix side. The overall reaction flux equation is given by:

$J_{CIV}= \frac{\frac{V_{maxf}}{K_{A}^{2}K_{B}^{0.5}}\left(\begin{matrix} \exp\left\{\beta_{IV}\left(\frac{-2\Delta G_{H}}{RT}-\frac{2F\Delta\Psi}{RT}-\frac{\text{Δ}_{r}G_{CIV}^{'0}}{RT}\right)\right\}A^{2}B^{0.5} \\ -\exp\left\{\left(\beta_{IV}-1\right)\left(\frac{-2\Delta G_{H}}{RT}-\frac{2F\Delta\Psi}{RT}-\frac{\text{Δ}_{r}G_{CIV}^{'0}}{RT}\right)\right\}C^{2} \end{matrix}\right)}{\left(1+\frac{A^{2}}{K_{A}^{2}}+\frac{C^{2}}{K_{C}^{2}}\right)\left(1+\frac{B^{0.5}}{K_{B}^{0.5}}\right)}$ (A36)

The model parameters in Table A17 were estimated by simultaneously fitting the solution of Eq A36 to the data in Fig A14 using the MATLAB “*fmincon*” optimization program package. The solid lines in Fig A14 are the fits of Eq A36 to the data.

**Fig A14.** **CIV activities as a function of the substrate cytochrome c and membrane potential (A), or oxygen (B) [17, 18].** In Fig A14A, CIV enzyme turnover numbers were measured under different fractions of reduced cytochrome c and membrane potentials. In Fig A14B, CIV reaction fluxes were measured under different oxygen concentrations.

**Table A17. CIV model kinetic parameters**

| **Parameters** | **Definition** | **Value** | **Source** |
| --- | --- | --- | --- |
| *V_maxf_* | Maximum forward reaction rate | 3.8 µmol/s/mg enzyme | Estimated using data in Fig A14 |
| *K_A_* | CytCr binding constant | $680\times10^{-6} M$ | Estimated using data in Fig A14 |
| *K_B_* | Oxygen binding constant | $5.4\times10^{-6} M$ | Estimated using data in Fig A14 |
| *K_C_* | CytCo binding constant | $680\times10^{-6} M$ | Assumed the same as K_A_ |
| *β_CIV_* | Complex IV free energy barrier | 0.5 | Fixed |
| $\text{Δ}_{r}G_{CIV}^{'0}$ | Gibbs free energy of the reaction (pH=7) | -122.94KJ/mol | [4] |

### Reaction 15: Complex V (CV)

### ADP_m_ + Pi_m_ +3H_i_ + H_m_^+^ ⇌ ATP_m_ + 3H_m_^+^

For this reaction, the reactant concentrations are denoted as A = $[ADP_{m}]$, B = $[Pi_{m}]$, C = $[ATP_{m}]$, and the participating co-factor pair is ADP/ATP. Thus, the overall reaction flux equation is given by:

$J_{CV}=\frac{\frac{V_{maxf}}{K_{A}K_{B}}\left\{\exp\left(\frac{3\beta_{V}(\Delta G_{H}-\text{Δ}_{r}G_{CV}^{'0})}{RT}\right)AB-\exp\left(\frac{3\left(\beta_{V}-1\right)(\Delta G_{H}-\text{Δ}_{r}G_{CV}^{'0})}{RT}\right)C\right\}}{\left(1+\frac{A}{K_{A}}+\frac{C}{K_{C}}\right)\left(1+\frac{B}{K_{B}}\right)}$ (A37)

Table A18. CV model kinetic parameters

| **Parameters** | **Definition** | **Value** | **Source** |
| --- | --- | --- | --- |
| *K_A_* | ADP binding constant | $10\times10^{-6} M$ | Fixed |
| *K_B_* | Pi binding constant | $0.5\times10^{-3} M$ | Fixed |
| *K_C_* | ATP binding constant | $1\times10^{-3} M$ | Fixed |
| $\beta_{CV}$ | Complex V free energy barrier | 0.5 | Fixed |
| $\text{Δ}_{r}G_{CV}^{'0}$ | Gibbs free energy of the reaction (pH=7) | 36.03KJ/mol | [4] |

## Metabolic transport fluxes across the mitochondrial inner membrane:

### Transport 1: Pyruvate-hydrogen cotransporter (PYRH)

PYR_e_ + H_e_^+^ ⇌ PYR_m_ + H_m_^+^

$J_{ PYRH}=\frac{T_{max}\left(\frac{[PYR_{e}][H_{e}^{+}]}{K_{PYR}K_{H}}-\frac{[PYR_{m}][H_{m}^{+}]}{K_{PYR}K_{H}}\right)}{1+\frac{[PYR_{e}]}{K_{PYR}}+\frac{[PYR_{m}]}{K_{PYR}}+\frac{[H_{e}^{+}]}{K_{H}}+\frac{[H_{m}^{+}]}{K_{H}}+\frac{[PYR_{e}]}{K_{PYR}}\frac{[H_{e}^{+}]}{K_{H}}+\frac{[PYR_{m}]}{K_{PYR}}\frac{[H_{m}^{+}]}{K_{H}}}$ (A38)

Table A19. PYRH model kinetic parameters

| **Parameters** | **Definition** | **Value** | **Source** |
| --- | --- | --- | --- |
| *K_PYR_* | PYR binding constant | $0.24\times10^{-3} M$ | [19] |
| *K_H_* | H^+^ binding constant | 1 $\times{10}^{-7}$ M | Fixed |

### Transport 2: Glutamate-hydrogen cotransporter (GLUH)

GLU_e_ + H_e_^+^ ⇌ GLU_m_ + H_m_^+^

$J_{GLUH}=\frac{T_{max}\left(\frac{\left[GLU_{e}\right]\left[H_{e}\right]}{K_{GLU}K_{H}}-\frac{\left[GLU_{m}\right]\left[H_{m}\right]}{K_{GLU}K_{H}}\right)}{1+\frac{\left[GLU_{e}\right]}{K_{GLU}}+\frac{\left[GLU_{m}\right]}{K_{GLU}}+\frac{\left[H_{e}\right]}{K_{H}}+\frac{\left[H_{m}\right]}{K_{H}}+\frac{\left[GLU_{e}\right]\left[H_{e}\right]}{K_{GLU}K_{H}}+\frac{\left[GLU_{m}\right]\left[H_{m}\right]}{K_{GLU}K_{H}}}$ (A39)

Table A20. GLUH model kinetic parameters

| **Parameter** | **Definition** | **Value** | **Source** |
| --- | --- | --- | --- |
| *K_GLU_* | GLU binding constant | $0.5\times10^{-3} M$ | Fixed |
| *K_H_* | Proton binding constant | $1\times10^{-7} M$ | Fixed |

### Transport 3&4: Dicarboxylate carrier (DCC)

| DCC (SUC): Pi_e_ + SUC_m_ ⇌ Pi_m_ + SUC_e_  DCC (MAL): Pi_e_ + MAL_m_ ⇌ Pi_m_ + MAL_e_ $J_{ DCC(MAL)}=\frac{T_{max}\left(\frac{\left[Pi_{e}\right]\left[MAL_{m}\right]}{K_{Pi}K_{MAL}}-\frac{\left[Pi_{m}\right]\left[MAL_{e}\right]}{K_{Pi}K_{MAL}}\right)}{\left(\begin{matrix} 1+\frac{\left[Pi_{m}\right]}{K_{Pi}}+\frac{\left[Pi_{e}\right]}{K_{Pi}}+\frac{\left[MAL_{m}\right]}{K_{MAL}}+\frac{\left[MAL_{e}\right]}{K_{MAL}}+\frac{\left[SUC_{m}\right]}{K_{SUC}}+\frac{\left[SUC_{e}\right]}{K_{SUC}}+ \\ \frac{\left[MAL_{e}\right]\left[Pi_{m}\right]}{K_{MAL}K_{Pi}}+\frac{\left[MAL_{m}\right]\left[Pi_{e}\right]}{K_{MAL}K_{Pi}}+\frac{\left[SUC_{e}\right]\left[Pi_{m}\right]}{K_{SUC}K_{Pi}}+\frac{\left[SUC_{m}\right]\left[Pi_{e}\right]}{K_{SUC}K_{Pi}} \end{matrix}\right)}$ (A40) $J_{ DCC(SUC)}=\frac{T_{max}\left(\frac{\left[Pi_{e}\right]\left[SUC_{m}\right]}{K_{Pi}K_{SUC}}-\frac{\left[Pi_{m}\right]\left[SUC_{e}\right]}{K_{Pi}K_{SUC}}\right)}{\left(\begin{matrix} 1+\frac{\left[Pi_{m}\right]}{K_{Pi}}+\frac{\left[Pi_{e}\right]}{K_{Pi}}+\frac{\left[MAL_{m}\right]}{K_{MAL}}+\frac{\left[MAL_{e}\right]}{K_{MAL}}+\frac{\left[SUC_{m}\right]}{K_{SUC}}+\frac{\left[SUC_{e}\right]}{K_{SUC}}+ \\ \frac{\left[MAL_{e}\right]\left[Pi_{m}\right]}{K_{MAL}K_{Pi}}+\frac{\left[MAL_{m}\right]\left[Pi_{e}\right]}{K_{MAL}K_{Pi}}+\frac{\left[SUC_{e}\right]\left[Pi_{m}\right]}{K_{SUC}K_{Pi}}+\frac{\left[SUC_{m}\right]\left[Pi_{e}\right]}{K_{SUC}K_{Pi}} \end{matrix}\right)}$ (A41) |
| --- |

Table A21. DCC model kinetic parameters

| **Parameter** | **Definition** | **Value** | **Source** |
| --- | --- | --- | --- |
| *K_Pi_* | Pi binding constant | $0.93\times10^{-3} M$ | [19] |
| *K_MAL_* | MAL binding constant | $1.17\times10^{-3} M$ | [19] |
| *K_SUC_* | SUC binding constant | $1\times10^{-3} M$ | Fixed |

### Transport 5: Tricarboxylate carrier (TCC)

TCC is non-electrogenic, only ${HCIT}^{2-}$ and ${MAL}^{2-}$ are accepted as transport species of TCC. One proton must bind to CIT for transport process to occur.

HCIT_e_ + MAL_m_ ⇌ HCIT_m_ + MAL_e_

The flux equation for this antiporter is:

$J_{TCC}=\frac{T_{ max}\left(\frac{\left[MAL_{e}\right]\left[H_{m}\right]\left[CIT_{m}\right]}{K_{CIT}K_{H}K_{MAL}}-\frac{\left[MAL_{m}\right]\left[H_{e}\right]\left[CIT_{e}\right]}{K_{CIT}K_{MAL}K_{H}}\right)}{\begin{matrix} 1+\frac{\left[CIT_{m}\right]\left[H_{m}\right]}{K_{CIT}K_{H}}+\frac{\left[CIT_{e}\right]\left[H_{e}\right]}{K_{CIT}K_{H}}+\frac{\left[MAL_{e}\right]}{K_{MAL}}+\frac{\left[MAL_{m}\right]}{K_{MAL}} \\ +\frac{\left[CIT_{m}\right]\left[H_{m}\right]\left[MAL_{e}\right]}{K_{CIT}K_{H}K_{MAL}}+\frac{\left[CIT_{e}\right]\left[H_{e}\right]\left[MAL_{m}\right]}{K_{CIT}K_{H}K_{MAL}} \end{matrix}}$ (A42)

Table A22. TCC model kinetic parameters

| **Parameter** | **Definition** | **Value** | **Source** |
| --- | --- | --- | --- |
| *K_CIT_* | CIT binding constant | $1\times10^{-3} M$ | Fixed |
| *K_MAL_* | MAL binding constant | $0.25\times10^{-3} M$ | [19] |
| *K_H_* | Proton binding constant | $1\times10^{-7} M$ | Fixed |

### Transport 6: AKG-MAL exchanger (OME)

AKG_e_ + MAL_m_ ⇌ AKG_m_ + MAL_e_

$J_{OME}=\frac{T_{max}\left(\frac{\left[MAL_{m}\right]\left[AKG_{e}\right]}{K_{AKG}K_{MAL}}-\frac{\left[AKG_{m}\right]\left[MAL_{e}\right]}{K_{AKG}K_{MAL}}\right)}{\left(\begin{matrix} 1+\frac{\left[MAL_{m}\right]}{K_{MAL}}+\frac{\left[MAL_{e}\right]}{K_{MAL}}+\frac{\left[AKG_{m}\right]}{K_{AKG}}+\frac{\left[AKG_{e}\right]}{K_{AKG}} \\ +\frac{\left[AKG_{m}\right]\left[MAL_{e}\right]}{K_{AKG}K_{MAL}}+\frac{\left[MAL_{m}\right]\left[AKG_{e}\right]}{K_{AKG}K_{MAL}} \end{matrix}\right)}$ (A43)

Table A23. OME model kinetic parameters

| **Parameter** | **Definition** | **Value** | **Source** |
| --- | --- | --- | --- |
| *K_AKG_* | AKG binding constant | $0.24\times10^{-3} M$ | [19]* |
| *K_MAL_* | MAL binding constant | $1\times10^{-3} M$ | [19]* |
| *K_H_* | Proton binding constant | $1\times10^{-7} M$ | Fixed |

*External binding constant and internal binding constant for AKG are reported to be 0.31 mM and 0.17 mM, respectively [19]. For simplicity, internal binding constant and external binding constant are assumed the same in this model. So an average value of 0.24 mM is used. Similarly, external binding constant and internal binding constants are 1.36 mM and 0.71 mM, respectively. Thus an average value of 1 mM is used as MAL binding constant.

### Transport 7: ASP-HGLU exchanger (GAE)

ASP_e_ + HGLU_m_ ⇌ ASP_m_ + HGLU_e_

$J_{GAE}=\frac{T_{max}\left\{\exp\left(\frac{0.5F\Psi}{RT}\right)\frac{[ASP_{e}][H_{m}][GLU_{m}]}{K_{ASP}K_{H}K_{GLU}}-\exp\left(\frac{-0.5F\Psi}{RT}\right)\frac{[ASP_{m}][H_{e}][GLU_{e}]}{K_{ASP}K_{H}K_{GLU}}\right\}}{\begin{matrix} 1+\frac{\left[H_{e}\right]\left[GLU_{e}\right]}{K_{H}K_{GLU}}+\frac{\left[H_{m}\right]\left[GLU_{m}\right]}{K_{H}K_{GLU}}+\frac{\left[ASP_{m}\right]}{K_{ASP}}+\frac{\left[ASP_{e}\right]}{K_{ASP}} \\ +\frac{\left[GLU_{e}\right]\left[H_{e}\right]\left[ASP_{m}\right]}{K_{GLU}K_{H}K_{ASP}}+\frac{\left[GLU_{m}\right]\left[H_{m}\right]\left[ASP_{e}\right]}{K_{GLU}K_{H}K_{ASP}} \end{matrix}}$ (A44)

Table A24. GAE model kinetic parameters

| **Parameter** | **Definition** | **Value** | **Source** |
| --- | --- | --- | --- |
| *K_ASP_* | ASP binding constant | $0.12\times10^{-3} M$ | [19] |
| *K_GLU_* | GLU binding constant | $0.25\times10^{-3} M$ | [19] |
| *K_H_* | Proton binding constant | $1\times10^{-7} M$ | Fixed |

### Transport 8: ATP-ADP anti-transporter (Adenine Nucleotide Translocase, ANT):

ADP_e_ + ATP_m_ ⇌ ADP_m_ + ATP_e_

Free ATP carries four negative charges while free ADP carries three negative charges. Therefore, the transport process catalyzed by ANT is affected by membrane potential. The flux equation was modified based on [2] to account for the effect of membrane potential. The transport flux ATP vs. ADP via ANT is given by:

$J_{ ANT}=\frac{T_{max}\left\{\exp\left(\frac{\beta_{ANT}F\Delta\Psi}{RT}\right)\frac{\left[ADP_{e}\right]\left[ATP_{m}\right]}{K_{ADP}K_{ATP}}-\exp\left(\frac{(\beta_{ANT}-1)F\Delta\Psi}{RT}\right)\frac{\left[ADP_{m}\right]\left[ATP_{e}\right]}{K_{ADP}K_{ATP}}\right\}}{\left(1+\frac{\left[ADP_{m}\right]}{K_{ADP}}+\frac{\left[ATP_{m}\right]}{K_{ATP}}\exp\left(\frac{\beta_{ANT}F\Delta\Psi}{RT}\right)\right)\left(1+\frac{\left[ADP_{e}\right]}{K_{ADP}}+\frac{\left[ATP_{e}\right]}{K_{ATP}}\exp\left(\frac{(\beta_{ANT}-1)F\Delta\Psi}{RT}\right)\right)}$ (A45)

Table A25. ANT model kinetic parameters

| **Parameters** | **Definition** | **Value** | **Source** |
| --- | --- | --- | --- |
| *K_ADP_* | ADP binding constant | $10\times10^{-6} M$ | [19] |
| *K_ATP_* | ATP binding constant | $10\times10^{-6} M$ | [19] |
| *β_ANT_* | ANT free energy barrier | 0.6 | [2] |

### Transport 9: Inorganic phosphate carrier (PIC)

Pi_e_ + H_e_^+^ ⇌ Pi_m_ + H_m_^+^

$J_{ PIC}=\frac{T_{max}\left(\frac{[Pi_{e}][H_{e}^{+}]}{K_{Pi}K_{H}}-\frac{[Pi_{m}][H_{m}^{+}]}{K_{Pi}K_{H}}\right)}{1+\frac{[Pi_{e}]}{K_{Pi}}+\frac{[Pi_{m}]}{K_{Pi}}+\frac{[H_{e}^{+}]}{K_{H}}+\frac{[H_{m}^{+}]}{K_{H}}+\frac{[Pi_{e}]}{K_{Pi}}\frac{[H_{e}^{+}]}{K_{H}}+\frac{[Pi_{m}]}{K_{Pi}}\frac{[H_{m}^{+}]}{K_{H}}}$ (A46)

Table A26. PIC model kinetic parameters

| **Parameters** | **Definition** | **Value** | **Source** |
| --- | --- | --- | --- |
| *K_Pi_* | Pi binding constant | $9.4\times10^{-3} M$ | [19] |
| *K_H_* | H^+^ binding constant | $1\times10^{-7} M$ | Fixed |

### Transport 10: Passive proton leak [2]

$J_{HLEAK}=\frac{T_{max}}{K_{H}}\left(\left[H_{i}^{+}\right]\exp\left(\frac{F\Delta\Psi}{2RT}\right)-\left[H_{m}^{+}\right]\exp\left(-\frac{F\Delta\Psi}{2RT}\right)\right)$ (A47)

Table A27. Proton leak model kinetic parameters

| **Parameters** | **Definition** | **Value** | **Source** |
| --- | --- | --- | --- |
| *K_H_* | H^+^ binding constant | $1\times10^{-7} M$ | Fixed |

## Part D: Governing mass balance equations for the integrated bioenergetics model

**Buffer region:**

$V_{e}\frac{d[Pi_{e}]}{dt}=-J_{PIC}+J_{DCC(MAL)}+J_{DCC(SUC)}$ (A48)

$V_{e}\frac{d[ADP_{e}]}{dt}=-J_{ANT}$ (A49)

$V_{e}\frac{d[ATP_{e}]}{dt}=J_{ANT}$ (A50)

$V_{e}\frac{d[PYR_{e}]}{dt}=-J_{PYRH}$ (A51)

$V_{e}\frac{d[MAL_{e}]}{dt}=-J_{OME}-J_{DCC(MAL)}-J_{TCC}$ (A52)

$V_{e}\frac{d[CIT_{e}]}{dt}=+J_{TCC}$ (A53)

$V_{e}\frac{d[AKG_{e}]}{dt}=+J_{OME}$ (A54)

$V_{e}\frac{d[SUC_{e}]}{dt}=-J_{DCC(SUC)}$ (A55)

$V_{e}\frac{d[GLU_{e}]}{dt}=-J_{GLUH}+J_{GAE}$ (A56)

$V_{e}\frac{d[ASP_{e}]}{dt}=-J_{GAE}$ (A57)

$\frac{d[H_{e}^{+}]}{dt}=0$ (A58)

**Inter-membrane space region:**

$\frac{d[H_{i}^{+}]}{dt}=0$ (A59)

$V_{i}\frac{d[CytC_{o}]}{dt}=-2J_{CIII}+2J_{CIV}$ (A60)

$\frac{d[CytC_{r}]}{dt}=-\frac{d[CytC_{o}]}{dt}$ (A61)

**Mitochondrial matrix region:**

$V_{m}\frac{d[GLU_{m}]}{dt}=J_{GOT}-J_{GAE}+J_{GLUH}$ (A62)

$V_{m}\frac{d[ASP_{m}]}{dt}=-J_{GOT}+J_{GAE}$ (A63)

$V_{m}\frac{d[PYR_{m}]}{dt}=-J_{PDH}+J_{PYRH}$ (A64)

$V_{m}\frac{d[OXA_{m}]}{dt}=J_{MDH}-J_{CITS}+J_{GOT}$ (A65)
 $V_{m}\frac{d[CIT_{m}]}{dt}=+J_{CITS}-J_{CITDH}-J_{TCC}$ (A66)

$V_{m}\frac{d[AKG_{m}]}{dt}=J_{CITDH}-J_{AKGDH}-J_{GOT}-J_{OME}$ (A67)

$V_{m}\frac{d[SCA_{m}]}{dt}=J_{AKGDH}-J_{SCAS}$ (A68)

$V_{m}\frac{d[SUC_{m}]}{dt}=J_{SCAS}-J_{SUC\text{DH}}+J_{DCC(SUC)}$ (A69)

$V_{m}\frac{d[FUM_{m}]}{dt}=J_{S\text{DH}}-J_{FH}$ (A70)

$V_{m}\frac{d[MAL_{m}]}{dt}=J_{FH}-J_{MDH}+J_{DCC(MAL)}+J_{TCC}+J_{OME}$ (A71)

$V_{m}\frac{d[FADH_{2,m}]}{dt}=J_{SDH}-J_{CII}$ (A72)

$V_{m}\frac{d[FAD_{m}^{+}]}{dt}=-V_{m}\frac{d[FADH_{2,m}]}{dt}$ (A73)

$V_{m}\frac{d[NADH_{m}]}{dt}=+J_{P\text{DH}}+J_{CITDH}+J_{AKGDH}+J_{MDH}-J_{CI}$ (A74)

$V_{m}\frac{d[NAD_{m}^{+}]}{dt}=-V_{m}\frac{d[NADH_{m}]}{dt}$ (A75)

$V_{m}\frac{d[ATP_{m}]}{dt}=-J_{ANT}+J_{NDK}+J_{CV}$ (A76)

$V_{m}\frac{d[ADP_{m}]}{dt}=-V_{m}\frac{d[ATP_{m}]}{dt}$ (A77)

$V_{m}\frac{d[GTP_{m}]}{dt}=J_{SCAS}-J_{NDK}$ (A78)

$V_{m}\frac{d[GDP_{m}]}{dt}=-V_{m}\frac{d[GTP_{m}]}{dt}$ (A79)

$V_{m}\frac{d[ACoA_{m}]}{dt}=J_{PDH}-J_{CITS}$ (A80)

$V_{m}\frac{d[CoA_{m}]}{dt}=-J_{P\text{DH}}+J_{CITS}+J_{SCAS}-J_{AKG\text{DH}}$ (A81)

$V_{m}\frac{d[Pi_{m}]}{dt}=J_{PIC}-J_{SCAS}-J_{CV}-J_{DCC(MAL)}-J_{DCC(SUC)}$ (A82)

$V_{m}\frac{d[H_{m}^{+}]}{dt}=+\frac{2.303}{\beta_{mit}}C_{m,H^{+}}\left(\begin{matrix} J_{HLEAK}-J_{GAE}-J_{TCC}+2J_{PIC}+J_{GLUH}+(1-2)J_{PDH} \\ +2J_{CITS}+J_{AKGDH}+J_{SCAS}+J_{MDH}-\left(4+1\right)J_{CI} \\ -2J_{CIII}-4J_{CIV}+\left(3-1\right)J_{CV} \end{matrix}\right)$ (A83)

$V_{m}\frac{d[UQ_{m}]}{dt}=-J_{CI}-J_{CII}+J_{CIII}$ (A84)

$V_{m}\frac{d[UQH_{2m}]}{dt}=-V_{m}\frac{d[UQ_{m}]}{dt}$ (A85)

**Mitochondria membrane potential:**

$\frac{d\Delta\Psi}{dt}=\frac{1}{C_{imm}}\left(4J_{CI}+2J_{CIII}+4J_{CIV}-3J_{ANT}-J_{HLEAK}-J_{ANT}\right)$ (A86)

where $\text{C}_{\text{imm}}$ is capacitance of the inner mitochondrial membrane.

**Oxygen consumption:**

For membrane potential experiments, the chamber is open to the atmosphere, and oxygen concentration is assumed to be constant. Therefore,

$\frac{d[O_{2}]}{dt}=0$ (A87)

For oximetry experiments, the chamber is a closed system, and oxygen is consumed by the mitochondria at complex IV. Therefore,

$\left(V_{m}+V_{e}+V_{i}\right)\frac{d[O_{2}]}{dt}=-0.5J_{CIV}$ (A88)

**References**

1. Pradhan RK, Vinnakota KC, Beard DA, Dash RK. Chapter 5 - Carrier-Mediated Transport Through Biomembranes. Transport in Biological Media. Boston: Elsevier; 2013. p. 181-212.

2. Wu F, Yang F, Vinnakota KC, Beard DA. Computer modeling of mitochondrial tricarboxylic acid cycle, oxidative phosphorylation, metabolite transport, and electrophysiology. J Biol Chem. 2007;282(34):24525-37. PubMed PMID: 17591785.

3. Dash RK, Li Y, Kim J, Beard DA, Saidel GM, Cabrera ME. Metabolic dynamics in skeletal muscle during acute reduction in blood flow and oxygen supply to mitochondria: in-silico studies using a multi-scale, top-down integrated model. PloS one. 2008;3(9):e3168. Epub 2008/09/10. doi: 10.1371/journal.pone.0003168. PubMed PMID: 18779864; PubMed Central PMCID: PMCPMC2526172.

4. Li Y, Lai N, Kirwan JP, Saidel GM. Computational Model of Cellular Metabolic Dynamics in Skeletal Muscle Fibers during Moderate Intensity Exercise. Cellular and molecular bioengineering. 2012;5(1):92-112. PubMed PMID: PMC3431029.

5. Bazil JN, Buzzard GT, Rundell AE. Modeling mitochondrial bioenergetics with integrated volume dynamics. PLoS computational biology. 2010;6(1):e1000632. Epub 2010/01/07. doi: 10.1371/journal.pcbi.1000632. PubMed PMID: 20052270; PubMed Central PMCID: PMCPMC2793388.

6. Tsai CS, Burgett MW, Reed LJ. Alpha-keto acid dehydrogenase complexes. XX. A kinetic study of the pyruvate dehydrogenase complex from bovine kidney. The Journal of biological chemistry. 1973;248(24):8348-52. Epub 1973/12/25. PubMed PMID: 4357736.

7. Beard DA, Vinnakota KC, Wu F. Detailed enzyme kinetics in terms of biochemical species: study of citrate synthase. PloS one. 2008;3(3):e1825. Epub 2008/03/20. doi: 10.1371/journal.pone.0001825. PubMed PMID: 18350161; PubMed Central PMCID: PMCPMC2266798.

8. Qi F, Chen X, Beard DA. Detailed kinetics and regulation of mammalian NAD-linked isocitrate dehydrogenase. Biochimica et biophysica acta. 2008;1784(11):1641-51. Epub 2008/08/02. doi: 10.1016/j.bbapap.2008.07.001. PubMed PMID: 18672100; PubMed Central PMCID: PMCPMC2584801.

9. Qi F, Pradhan RK, Dash RK, Beard DA. Detailed kinetics and regulation of mammalian 2-oxoglutarate dehydrogenase. BMC biochemistry. 2011;12:53. Epub 2011/09/29. doi: 10.1186/1471-2091-12-53. PubMed PMID: 21943256; PubMed Central PMCID: PMCPMC3195097.

10. Li X, Wu F, Beard DA. Identification of the kinetic mechanism of succinyl-CoA synthetase. Bioscience reports. 2013;33(1):145-63. Epub 2012/10/24. doi: 10.1042/bsr20120069. PubMed PMID: 23088689; PubMed Central PMCID: PMCPMC3549575.

11. Zeijlemaker WP, Dervartanian DV, Veeger C, Slater EC. Studies on succinate dehydrogenase. IV. Kinetics of the overall reaction catalysed by preparations of the purified enzyme. Biochimica et biophysica acta. 1969;178(2):213-24. Epub 1969/04/22. PubMed PMID: 5814428.

12. Heyde E, Ainsworth S. Kinetic studies on the mechanism of the malate dehydrogenase reaction. The Journal of biological chemistry. 1968;243(9):2413-23. Epub 1968/05/10. PubMed PMID: 4296842.

13. Dash RK, Qi F, Beard DA. A Biophysically Based Mathematical Model for the Kinetics of Mitochondrial Calcium Uniporter. Biophysical Journal. 2009;96(4):1318-32. doi: 10.1016/j.bpj.2008.11.005. PubMed PMID: PMC2717240.

14. Dash RK, Beard DA. Analysis of cardiac mitochondrial Na(+)–Ca(2+) exchanger kinetics with a biophysical model of mitochondrial Ca(2+) handing suggests a 3: 1 stoichiometry. The Journal of physiology. 2008;586(Pt 13):3267-85. doi: 10.1113/jphysiol.2008.151977. PubMed PMID: PMC2538784.

15. Bazil JN, Pannala VR, Dash RK, Beard DA. Determining the origins of superoxide and hydrogen peroxide in the mammalian NADH:ubiquinone oxidoreductase. Free radical biology & medicine. 2014;77:121-9. Epub 2014/09/23. doi: 10.1016/j.freeradbiomed.2014.08.023. PubMed PMID: 25236739; PubMed Central PMCID: PMCPMC4258523.

16. Bazil Jason N, Vinnakota Kalyan C, Wu F, Beard Daniel A. Analysis of the Kinetics and Bistability of Ubiquinol:Cytochrome c Oxidoreductase. Biophysical Journal. 2013;105(2):343-55. doi: 10.1016/j.bpj.2013.05.033. PubMed PMID: PMC3714890.

17. Murphy MP, Brand MD. The control of electron flux through cytochrome oxidase. Biochemical Journal. 1987;243(2):499-505. PubMed PMID: PMC1147883.

18. Wilson DF, Rumsey WL, Green TJ, Vanderkooi JM. The oxygen dependence of mitochondrial oxidative phosphorylation measured by a new optical method for measuring oxygen concentration. The Journal of biological chemistry. 1988;263(6):2712-8. Epub 1988/02/25. PubMed PMID: 2830260.

19. Palmieri F. Mitochondrial carrier proteins. FEBS letters. 1994;346(1):48-54. doi: <https://doi.org/10.1016/0014-5793(94)00329-7>.
